# Perplexing Polyphenolics: The Isolations, Syntheses, Reappraisals, and Bioactivities of Flavonoids, Isoflavonoids, and Neoflavonoids from 2016 to 2022

**DOI:** 10.3390/life13030736

**Published:** 2023-03-09

**Authors:** Syed Muhammad Umer, Shahbaz Shamim, Khalid Mohammed Khan, Rahman Shah Zaib Saleem

**Affiliations:** 1 Department of Chemistry and Chemical Engineering, SBASSE, Lahore University of Management Sciences, Sector-U, DHA, Lahore 54792, Pakistan; 2 H.E.J. Research Institute of Chemistry, International Center for Chemical and Biological Sciences, University of Karachi, Karachi 75270, Pakistan; 3 Department of Clinical Pharmacy, Institute for Research and Medical Consultations (IRMC), Imam Abdulrahman Bin Faisal University, Dammam P.O. Box 31441, Saudi Arabia

**Keywords:** flavonoids, isoflavonoids, neoflavonoids, total synthesis, semisynthesis, bioactivities, reappraisals

## Abstract

Flavonoids, isoflavonoids, neoflavonoids, and their various subcategories are polyphenolics–an extensive class of natural products. These compounds are bioactive and display multiple activities, including anticancer, antibacterial, antiviral, antioxidant, and neuroprotective activities. Thus, these compounds can serve as leads for therapeutic agents or targets for complex synthesis; they are coveted and routinely isolated, characterized, biologically evaluated, and synthesized. However, data regarding the compounds’ sources, isolation procedures, structural novelties, bioactivities, and synthetic schemes are often dispersed and complex, a dilemma this review aims to address. To serve as an easily accessible guide for researchers wanting to apprise themselves of the latest advancements in this subfield, this review summarizes seventy-six (76) articles published between 2016 and 2022 that detail the isolation and characterization of two hundred and forty-nine (249) novel compounds, the total and semisyntheses of thirteen (13) compounds, and reappraisals of the structures of twenty (20) previously reported compounds and their bioactivities. This article also discusses new synthetic methods and enzymes capable of producing or modifying flavonoids, isoflavonoids, or neoflavonoids.

## 1. Introduction

Natural products—compounds isolated from natural sources—and their derivatives represent an immense segment of modern medicine: approximately 25% of all Food and Drug Administration (FDA) and European Medical Agency (EMA) approved drugs are plant-based [1]. Additionally, a third of FDA-approved drugs between 1990 and 2010 were either natural products or a derivative of them [2]. Furthermore, the World Health Organization’s list of essential medicines features more than twenty-two (22) compounds sourced exclusively from angiosperms [3]. Another estimate lists 40% of all available medicines as either natural products or derivatives [4,5].

Plants carry a massive reservoir of secondary metabolites that are potent and bioactive molecules. Millions of years of evolutionary pressures have fine-tuned these molecules to serve as effective defenses against both endogenous and exogenous threats, boosting their host’s survivability and explaining their potencies as antiviral, antibacterial, and anticancer agents [6,7,8]. Apart from their biological activities, natural products, under their structural complexity, are coveted targets in synthesis. Their novel structures help chemists to explore new chemical spaces and drive the search for new synthetic strategies, new methods of functionalizing chemical bonds, catalyst design, and inspired synthetic analogs [9,10]. However, only a small fraction—five to fifteen percent—of terrestrial plant species have been analyzed for their secondary metabolite content. Additionally, the microbial domain, which accounts for ninety percent of all natural diversity, has only barely (<1%) been explored [11]. The trove of species whose chemical contents have not yet been characterized represents a major opportunity for researchers to explore and analyze further, uncovering potent, unprecedented compounds in the process [12].

Natural products are grouped into four broad categories: polyphenolics, polyketides, terpenoids, and alkaloids [13,14]. Polyphenolics are defined as natural products containing multiple phenol units and are ubiquitous in plants. Additionally, they are important antioxidants (boosting their potential therapeutic use against cancer, cardiovascular, and neurodegenerative diseases), in addition to modulating several other biological targets [15,16]. Polyphenolics are further subdivided into four major classes: phenolic acids, stilbenes, lignans, and flavonoids, with the latter class containing more than 6000 characterized compounds, including therapeutics such as rutin, crofelemer, and rotenone [17,18].

This review summarizes recent discoveries—isolations, structural novelties, syntheses, bioactivities—vis-à-vis flavonoids, isoflavonoids, and neoflavonoids so that it can serve as a convenient, understandable resource for interested researchers. SciFinder was used as the primary database. The keywords to identify novel isolates were “flavonoid,” “novel,” and “isolation.” Similarly, the keywords to search for synthetic methods were “flavonoid” and “synthesis.” The search was restricted to only include papers published from 2016 to 2022.

For clarity, the ‘flavonoid’ class was defined to include flavones, flavonols, flavans, flavanones, flavanols, and flavanonols only. As mentioned above, isoflavonoids and neoflavonoids were defined to include their iso- and neo-variants of the subclasses. This search yielded sixty-one (61) articles detailing the isolations, characterizations, and biological evaluations of two-hundred and forty-nine (249) polyphenolic compounds. In addition, ten (10) articles describing the synthesis of twenty-one (21) flavonoids or isoflavonoids were also included.

Of the compounds isolated, most were flavonoids, followed by isoflavonoids—only one neoflavonoid was found. These compounds displayed anticancer, antibacterial, anti-inflammatory, antifungal, antiparasitic, and antiviral activities. The compounds also exhibited inhibition of targets such as protein tyrosine phosphatase 1B, tyrosinase, and α-glucosidase.

## 2. Isolation of Novel Flavonoids, Isoflavonoids, and Neoflavonoids

### 2.1. Source Analysis

**Figure 1:**
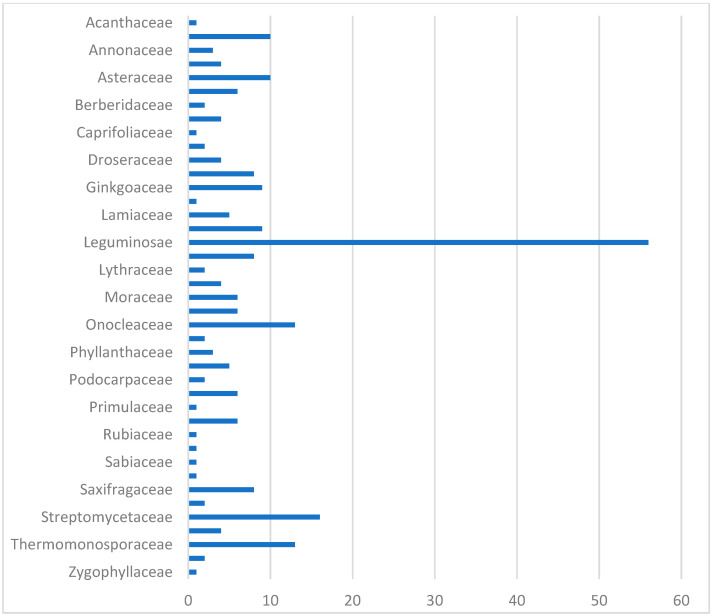
Natural sources of compounds **1–249**.

As Figure 1 shows, an overwhelming majority (73%) of the novel compounds isolated were sourced from flowering plants. Fifty-six (56) compounds were produced by the *Leguminosae* family, while *Amaranthaceae* and *Asteraceae* yielded ten (10) isolates each. The other one hundred and five (105) compounds were sourced from thirty-one (31) angiosperm families. Additionally, thirty-nine (39) compounds were sourced from non-flowering plants: thirteen (13) were isolated from *Onocleaceae* and nine (9) from *Ginkgoaceae* species. Furthermore, twenty-nine (29) compounds were isolated from bacteria, with *Streptomycetaceae* producing sixteen (16) isolates and *Thermomonosporaceae* yielding the rest. No reports of novel flavonoids, isoflavonoids, or neoflavonoids isolated from tunicates, marine sponges, or fungi were included.

### 2.2. Isolation Methods

In this review, articles were selected from 2016 to 2022, and no novel method of isolating flavonoids, isoflavonoids, or neoflavonoids from mixtures extracted from natural sources was discussed, as apparent in Table 1. Conventional procedures involve extracting the source, often dried plant material, with a polar solvent, usually ethanol, ethyl acetate, or methanol. Next, the mixture is filtered, and the filtrate (crude extract) is concentrated and partitioned with organic solvents. Nonpolar solvents such as petroleum ether or n-hexane remove fatty acids. Finally, column chromatography separates and purifies polar phases to yield pure compounds.

### 2.3. Structural Novelties

**Figure 2:**
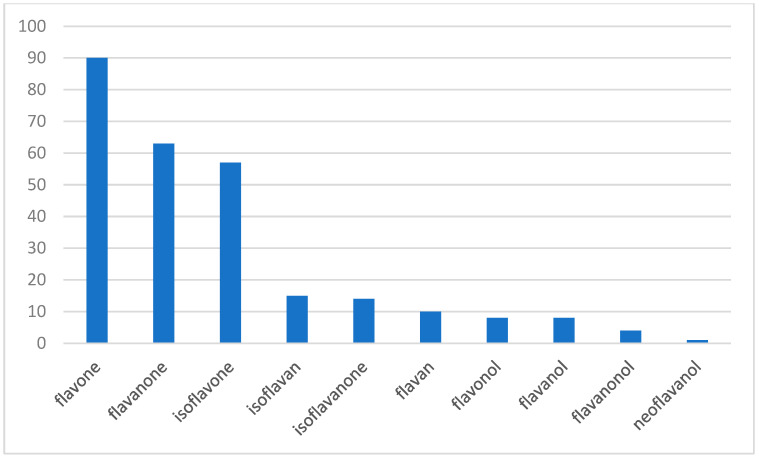
Occurrence of various subclasses in compounds **1–249**.

The presence of various subclasses in the isolated compounds is listed in Figure 2. As shown, flavonoid subunits were the most common, followed by isoflavonoid subunits. Only one example of a neoflavonoid (a neoflavanol) was found.

**Figure 3:**
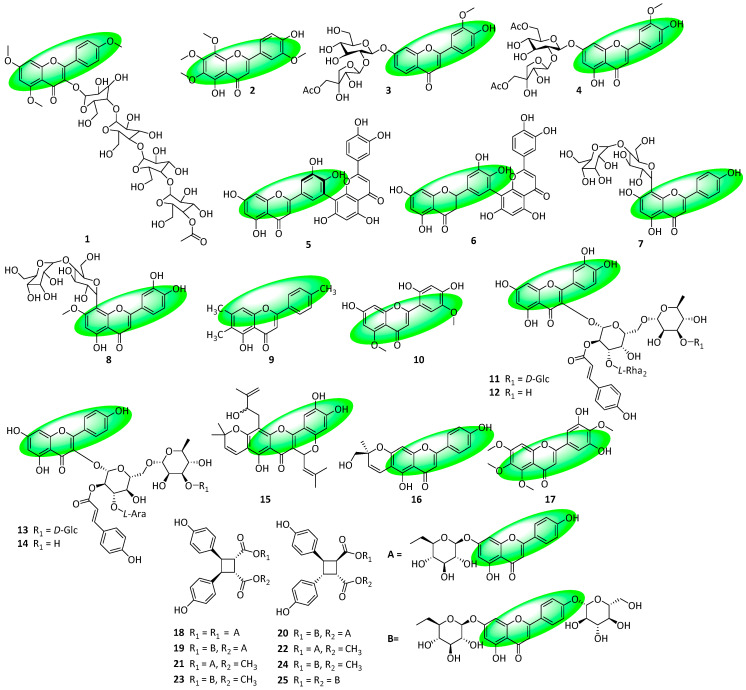
Structures of compounds **1–25**.

Compound **1** was isolated from the roots, stem, leaves, and flowers of *Strobilanthes kunthianus* and is a flavone glycoside (Figure 3) [19]. Similarly, compound **2** is also a flavone and was isolated from the aerial parts of *Adenosma bracteosum* [20]. Compounds **3** and **4** were isolated from the aerial parts and roots of *Glandularia selloi* and are glycosylated derivatives of chrysoeriol. They are acylated disaccharides and are the first acylated flavone *O*-glycosides to be isolated from the Verbenaceae family [21]. Compounds **5** and **6** were isolated from the gametophytes of *Ceratodon purpureus* and were fully characterized for the first time. Both compounds are dimers, with compound **5** containing two flavone subunits and compound **6** containing one flavone and one flavanone subunit [22]. The flavone C-glycosides, compounds **7** and **8**, were isolated from the leaves of *Afrocarpus gracilior* [23]. A trimethylated flavone, compound **9**, was extracted by analyzing the entire *Wulfenia amherstiana* plant [24]. Compound **10**, a methoxylated flavone, contains a 2′,4′,5′-trisubstituted B ring, a motif rarely encountered in nature. It was isolated from the stems and leaves of *Lonicera hypoglauca* [25]. Compounds **11–14** were isolated from Fuzhuan brick tea, produced from the leaves of *Camellia sinensis*. Compounds **11** and **12** represent quercetin acyl glycosides, while **13** and **14** are kaempferol acyl glycosides [26]. Compounds **15–16** were isolated from the twigs of *Artocarpus nigrifolius* and are prenylated flavones [27]. Compound **17** is a tetramethoxylated flavone isolated from the leaves and twigs of *Murraya tetramera* [28]. Compounds **18–25** are flavone glucoside cyclodimers and were isolated by analyzing the whole *Palhinhaea cernua* herb; they are the first flavonoid glucoside cyclodimers to be isolated from the *Lycopodiaceae* family. They also possess a unique cyclobutane ring and are truxinate esters: compounds **18**, **19**, **21**, and **23** are β-truxinates, while compounds **20**, **22**, **24**, and **25** are μ-truxinates. Furthermore, compounds **19** and **20**, **21** and **22**, and **23** and **24** represent three pairs of stereoisomers [29].

**Figure 4:**
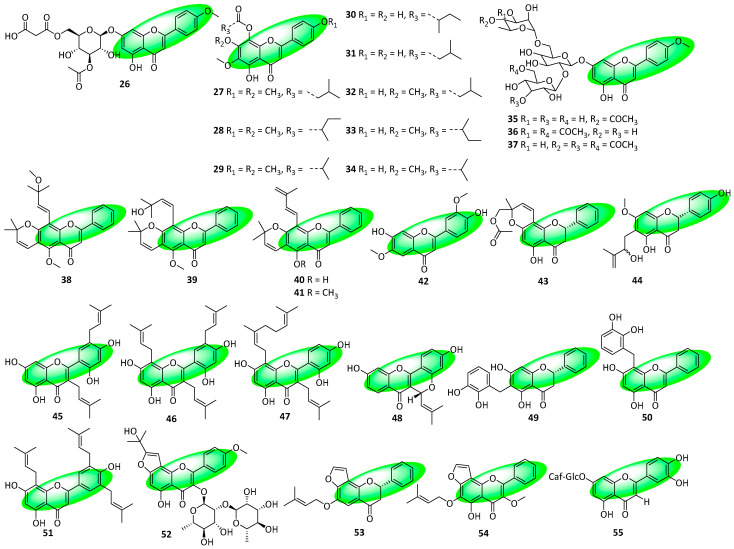
Structures of compounds **26–55**.

Compound **26**, a flavone glucoside, was isolated from the aerial parts of *Agastache rugosa* (Figure 4.) [30]. Compounds **27–34** are flavones that were isolated from the leaves of *Celmisia visc*osa. Their *O*-acylations are noteworthy as flavonoids with acylated core phenols are rare. Additionally, compound **31** contains a naturally occurring 3-methylbutanoate moiety, an unprecedented discovery. Similarly, flavonoids with 2-methylbutanoate and 2-methylpropanoate substituents are rare, giving compounds **30**, **27**, and **32** particular importance [31]. Compounds **35–37** are acetylated flavone glycosides isolated from the aerial parts of *Elsholtzia ciliata* [32]. Compounds **38–44** were isolated from the aerial parts of *Tephrosia linearis*. Compounds **38–41** contain a fused pyran-flavone core, and compounds **43** and **44** are flavanones [33]. Compounds **45–48**, all prenylated flavones, were isolated from the twigs of *Morus nigra* [34]. Compounds **49** and **50**, a flavanone and flavone, respectively, are chamanetin derivatives and feature C-benzylation. They were isolated from the leaves, stem, and root bark of *Sphaerocoryne gracilis* [35]. Compounds **51** and **52** are prenylated flavones isolated from the aerial parts of *Epimedium brevicornum*. The latter also contains a fused furan ring [36]. Compounds **53** and **54**, a furanoflavanone and a furanoflavone, respectively, were isolated from the vine stems of *Millettia velutina* [37]. Compound **55** is an acylated flavonoid glucoside that was isolated from the aerial parts of *Onopordum alexandrinum* [38]. 

**Figure 5:**
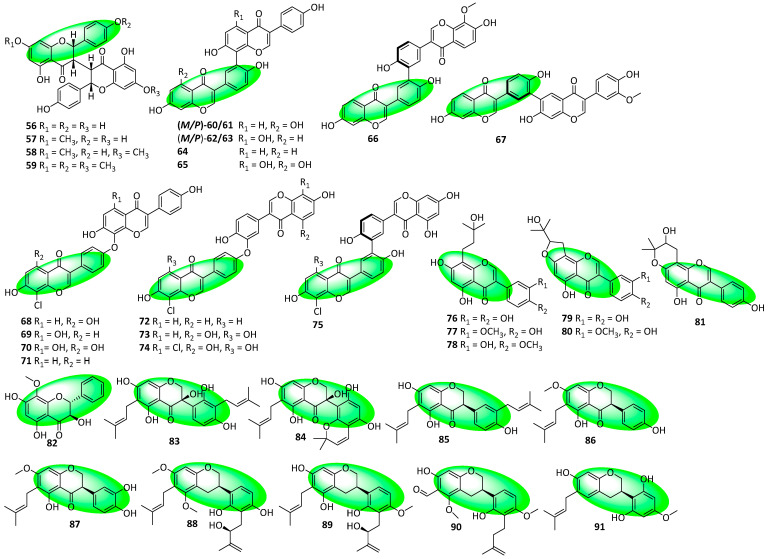
Structures of compounds **56–91**.

Compounds **56–59** are (3,3″)-linked biflavanone *O*-methyl ethers and were isolated from the bark of *Ouratea spectabilis*. Compounds **57**, **58**, and **59** are mono-, bi-, and trimethoxylated, respectively (Figure 5) [39]. Compounds **60–75** are biisoflavones that were isolated from *Streptomyces* sp. HDN154127, a Takla Makan desert-derived strain. Their dimeric and chlorinated forms are rare, and an actinomycete producing both features in an isoflavone has not been reported earlier. Compounds **60–65** are the first biisoflavone atropisomers isolated from a bacterial culture [40]. Compounds **76–81** are isoflavones isolated from the leaves of *Vatairea guianenis*. They all feature C8 prenylation, with compounds **76–78** displaying chain prenylation and compounds **79–81** displaying ring-closed prenylation [41]. Compound **82** is a flavanonol isolated from Dietes bicolor’s leaves. It features tetrasubstitution and has a fully oxygenated A-ring with an unsubstituted B-ring, rare motifs [42]. Compounds **83–91** are prenylated isoflavonoids isolated from the aerial parts of *Glycyrrhiza uralensis*. Compounds **83–87** are isoflavanones, while compounds **88–91** are isoflavans. Additionally, compound **90** contains a formyl group at C-6, a rarity among flavonoids and isoflavonoids [43]. 

**Figure 6:**
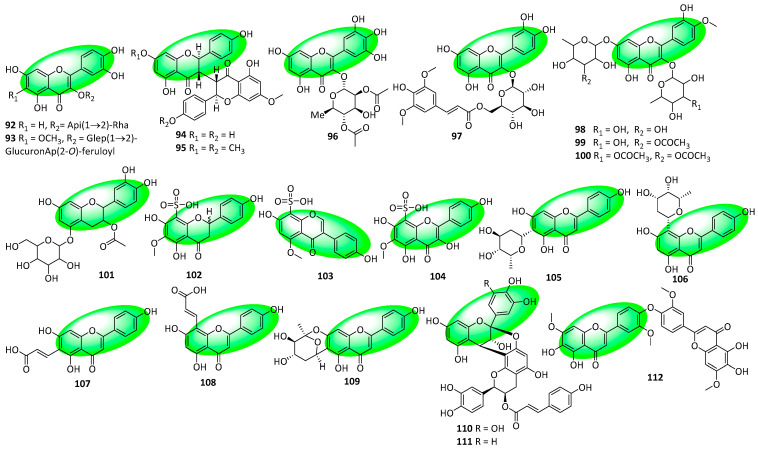
Structures of compounds **92–112**.

Compound **92** was isolated from the leaves of *Sabia limoniacea*. It is a flavone disaccharide and a quercetin derivative (Figure 6) [44]. Compound **93** is a flavone glucoside derivative isolated from the aerial parts of *Atriplex tatarica* [45]. Compounds **94** and **95** are biflavanones isolated from the stem bark of *Ochna holstii*. Compound **95** is a methoxy derivative of **94**, consistent with the *O*-methylation present in flavonoids [46]. Compound **96** was isolated from the shoots of *Myrsine africana* and is a flavone featuring five hydroxyl substitutions on its core [47]. Compound **97** is a glycosylated quercetin derivative and was isolated from the leaves of *Tetraena mongolica* [48]. Compounds **98–101** were isolated from the leaves of *Drosera magna*. Compounds **98–100** are flavone diglycosides, while compound **101** is a flavan glycoside [49]. Compounds **102–104** were isolated from the roots of *Phyllanthus acidus*. Compounds **102** and **103** are the first sulfonic acid-containing flavanone and isoflavone to be reported, respectively. Additionally, compound **104** is a sulfonic acid-containing flavonol [50]. Compounds **105–109** are flavones that were isolated from the leaves of *Mimosa caesalpiniifolia*. They are apigenin derivatives [51]. Compounds **110** and **111** were isolated from the leaves of *Ormosia arborea*. They are A-type proanthocyanidins—containing two flavan subunits—linked to a p-coumaroyl unit [52]. Compound **112** is a biflavone that was isolated from the aerial parts of *Salvia circinate* [53]. 

**Figure 7:**
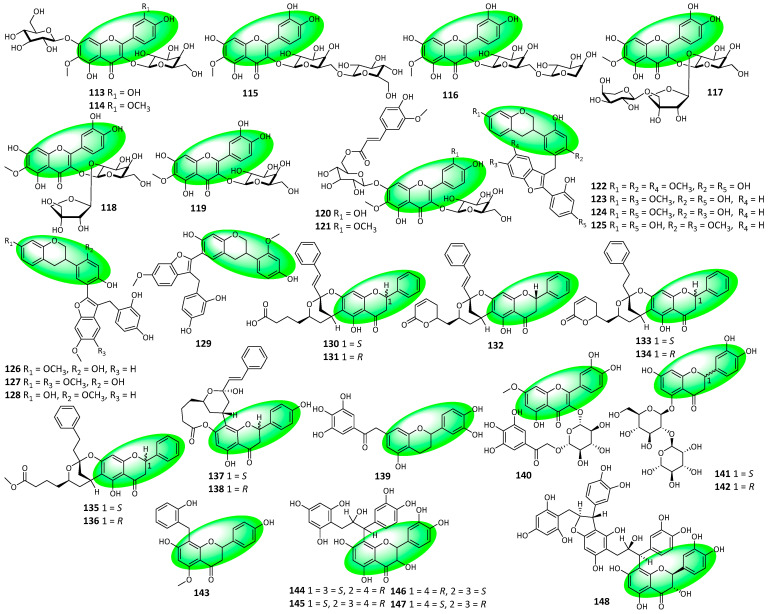
Structures of compounds **113–148**.

Compounds **113–121** are flavones isolated from the aerial parts of *Corispermum marschallii*. Compounds **113**, **115–118**, and **120** are patuletin glycosides, while compounds **114**, **119**, and **121** are spinacetin glycosides (Figure 7). Additionally, patulein glycosides previously isolated from the *Amaranthaceae* family only featured 3-*O*-glucosides, not 3-*O*-galactosides. This rarer substitution is present in compound **113** [54]. Compounds **122–129** are isoflavans isolated from the Brazilian Red Propolis; bees that produce the propolis feed on *Dalbergia ecastophyllum*. They all contain a benzofuran moiety [55]. Compounds **130–138** are complex flavanones and were isolated from the leaves and twigs of *Cryptocarya metcalfiana* [56]. Compounds **139** and **140** were isolated from the leaves of *Woodfordia uniflora*. Compound **139** is a mixture of two enantiomeric flavans, while compound **140** is a flavone and a quercetin derivative [57]. Compounds **141** and **142** were isolated from the shoots of *Cyclopia genistoides* and were completely characterized. They are flavanones and represent a pair of diastereomeric naringenin derivatives [58]. Compound **143** is a flavanone isolated from the fruits and leaves of *Melodorum siamensis* [59]. Compounds **144–148** are flavans isolated from the inner bark of *Pinus massoniana*. Compounds **144–147** are seco B-type procyanidin dimers, with compounds **144** and **145** optical antipodes of gambiriin A1 and A2, respectively. Similarly, compounds **146** and **147** are a pair of optical antipodes, while compound **148** is the first seco B-type procyanidin trimer [60]. 

**Figure 8:**
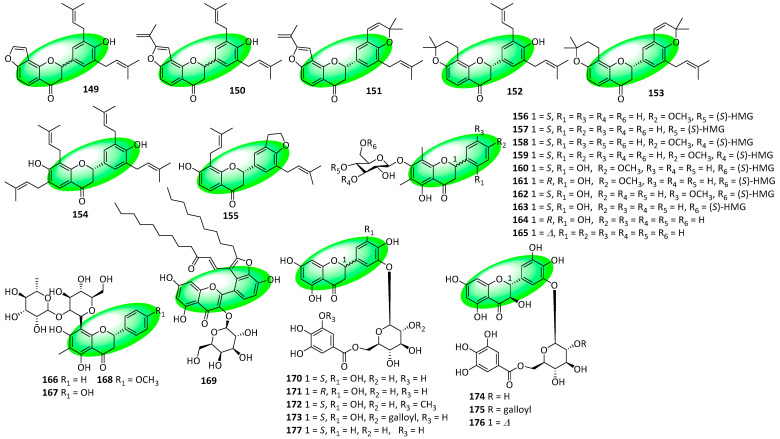
Structures of compounds **149–177**.

Compounds **149–155** are prenylated flavanones isolated from the roots and rhizomes of *Sophora tonkinensis* [61]. Compounds **156–168** are C-methylated flavanone glycosides isolated from the rhizomes of *Pentarhizidium orientale* (Figure 8). Compounds **156–163** are matteuorienates A–C analogs and contain a characteristic 3-hydroxy-3-methylglutaryl (HMG) moiety [62]. Compound **169** is a flavone isolated from the aerial parts of *Houttuynia cordata*. It is the first houttuynoid containing a bis-houttuynin chain connected to a flavonoid core to be reported [63]. Compounds **170–177** are flavonoid-based 3′-*O*-β-D-glucopyranosides with an acylated glucopyranosyl moiety. They were isolated from the aerial parts of *Saxifraga spinulosa*. Additionally, compounds **170–173** and **177** are flavanones, compounds **174** and **175** are flavanonols, and compound **176** is a flavonol [64]. 

**Figure 9:**
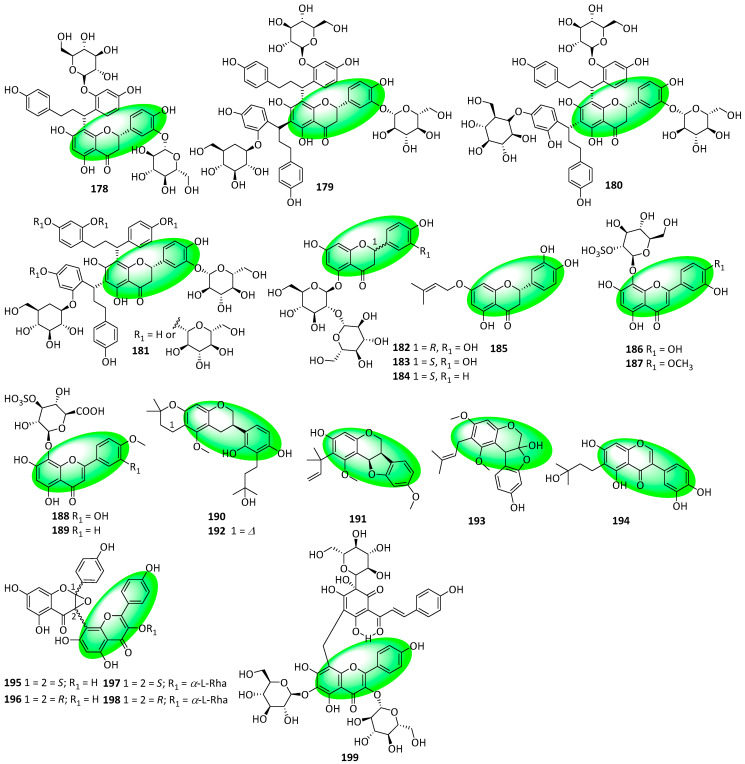
Structures of compounds **178** to **199**.

Compounds **178–181** contain glucosylated flavanones containing 1,3-diaryl propane C6–C3–C6 units (Figure 9). They were isolated from the fruits of *Mansoa hirsute*. Additionally, compound **178** is diglucosylated, while compounds **179–181** are triglucosylated and are isomeric with mansoin A [65]. Compounds **182–184** are flavanone 5-*O*-diglycosides that were isolated from the unripe fruits of *Berchemia berchemiifolia*. Compounds **182** and **183** are diastereomers and eriodictyol derivatives, while **184** is a naringenin derivative [66]. Compound **185** is prenylated flavanone and was isolated from the aerial parts of *Arcytophyllum thymifolium*. It is also an eriodictyol derivative [67]. Compounds **186–189** are flavones isolated from the roots of *Althaea officinalis*. They are hypolaetin-*O*-sulfoglycosides [68]. Compounds **190–194** were isolated from the roots and rhizomes of *Glycyrrhiza uralensis*. Compounds **190–192** are isoflavans; compound **193** is a neoflavanol, and compound **194** is an isoflavone [69]. Compounds **195–198** were isolated from the white petals of *Impatiens balsamina*. They are biflavonoid glycosides containing an isoflavanone and flavone subunit [70]. However, the initially reported epoxide motif was reappraised and replaced with a fused dihydrofuran motif (compounds **438–441**) [79]. Compound **199** is a quinochalcone C-glycoside containing a flavone linked by a methylene bridge. It was isolated from the florets of *Carthamus tinctorius*, which is the only known source of the extremely rare C-glycosylated quinochalcones [71]. 

**Figure 10:**
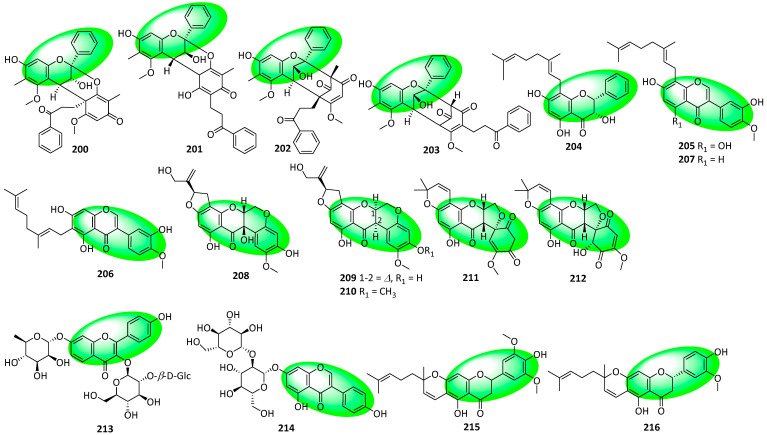
Structures of compounds **200–216**.

Compounds **200–203** are A-type flavanol-dihydroretrochalcone dimers isolated from the resin of *Daemonorops draco* (Figure 10) [72]. Compounds **204–214** were isolated from the fruits of *Amorpha fruticosa*. Compound **204** is a geranylated flavanonol, while compounds **205** to **207** are geranylated isoflavones. Additionally, compounds **208–212** are rotenoids and contain an isoflavanone core. Furthermore, compound **213** is a flavone glycoside, while compound **214** is an isoflavone glycoside [73]. Compounds **215** and **216** are geranylated flavanones and were isolated from the fruits of *Paulownia tomentosa* [74]. 

**Figure 11:**
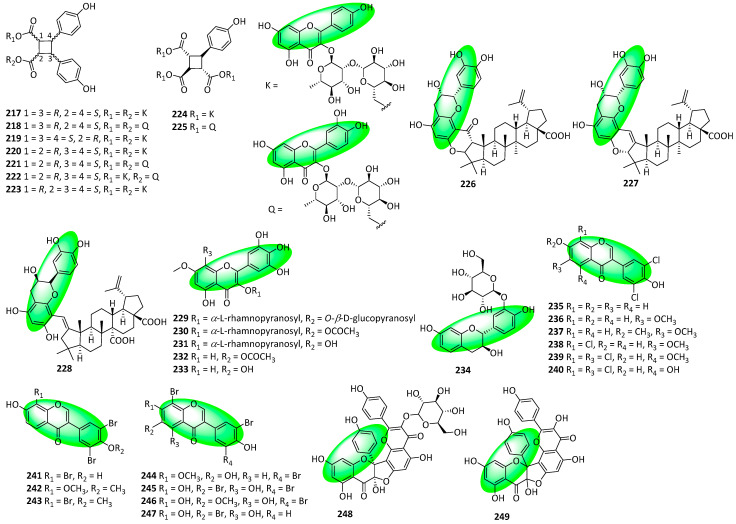
Structures of compounds **217–249**.

Compounds **217–225** are flavone-containing glycoside cyclodimers isolated from the leaves of *Ginkgo biloba*. Compounds **217–223** are truxinates—resembling compounds **18–25**—while compounds **224** and **225** are truxillates (Figure 11). Additionally, these compounds also contain a cyclobutane ring [75]. Compounds **226–228** are flavanol-containing compounds isolated from the roots of *Zizyphus jujuba*. Additionally, they are ceanothane-type triterpenoids bonded to a catechin moiety via carbon–carbon bonds; the first reported natural products to have a carbon–carbon bond between a triterpene and a flavonoid. Furthermore, the C2 carbonyl in compound **226** is characteristic of ceanothane-type triterpenoids, and likely directed the formation of the unique carbon–carbon bonds between the triterpene and flavonoid moieties [76]. Compounds **229–234** were isolated from the aerial parts of *Atraphaxis frutescens*. Compounds **229–234** are 7-methoxyflavonols containing a pyrogallol B-ring, with **230** and **232** being 8-*O*-acetyl derivatives of **231** and **233**, respectively. Additionally, compound **234** is a fisetinidol glucoside and has a flavanol core [77]. Compounds **235–247** were isolated from *Actinomadura* sp. RB99, which itself was extracted from the fungus-farming termites, *Macrotermes natalensis*. The isoflavones either display polychlorination, as in compounds **235–240,** or polybromination, as in compounds **241–247** [78]. Compounds **248** and **249** are biflavonoid glycosides and contain a flavanone and flavone unit. They have fused dihydrofuran rings akin to the reappraised structures of compounds **195–198** and were also isolated from the same source (white petals of *Impatiens balsamina*) [79]. 

## 3. Synthesis of Flavonoids and Isoflavonoids

### 3.1. Total Synthesis

The first total synthesis of neocyclomorusin (**278**) was completed in 2022. Compound **278** is a bioactive pyranoflavone, exhibiting cytotoxic, antioxidant, anti-inflammatory, and cholinesterase-inhibiting properties, and is mainly isolated from plants in the *Moraceae* family. In addition, other structurally related flavones, such as oxyisocyclointegrin (**264**), morusin (**276**), and cudraflavone B (**277**), were also synthesized. The synthetic route yields these flavones utilizing a Friedel–Crafts reaction, a Baker–Venkataraman (BK–VK) rearrangement, a selective epoxidation, and a novel S_N_2-type cyclization.

**Figure 12:**
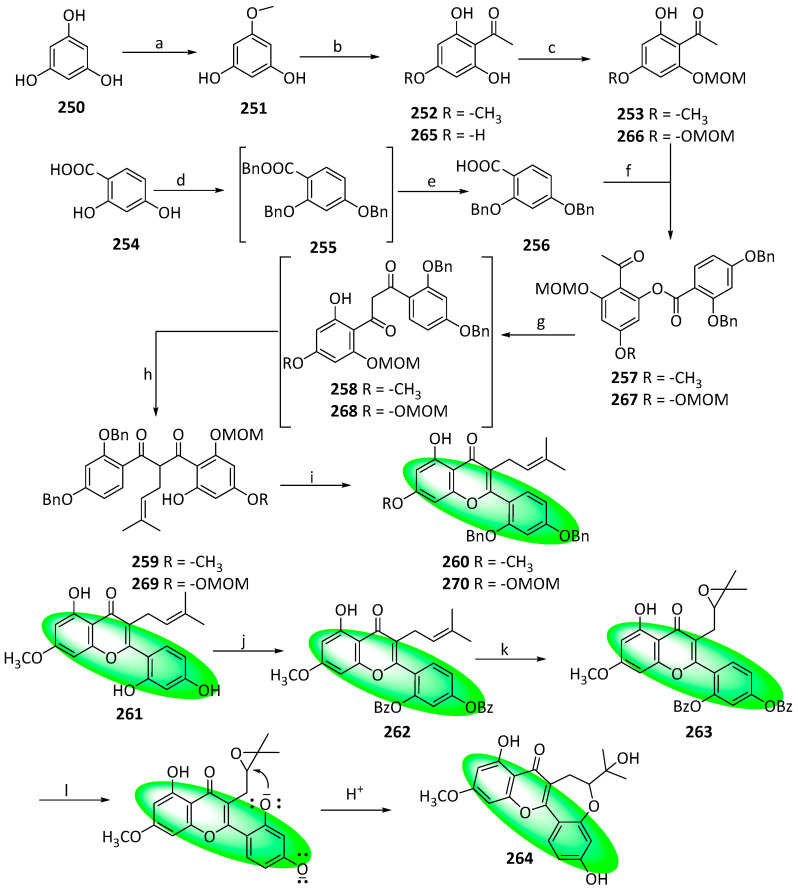
Synthesis of compound **264**; (**a**) CH_3_OH, H_2_SO_4_, rt, 12 h, 55%; (**b**) CH_3_COCl, AlCl_3_, CH_2_Cl_2_, rt, 12 h, **252**: 77%, **265**: 87%; (**c**) DIPEA, CH_2_Cl_2_, rt, 4 h, **253**: 2 eq of MOMBr, 80%, **260**: 4 eq of MOMBr, 70%; (**d**) BnBr, K_2_CO_3_, acetone, 65 °C, 18 h; (**e**) 5M NaOH, CH_3_OH, 75 °C, 4 h, 96% in two steps; (**f**) EDCI, DMAP, CH_2_Cl_2_, rt, 24 h, **257**: 84%, **267**: 86%; (**g**) NaH, DMSO, rt; (**h**) 3,3-dimethylallyl bromide, K_2_CO_3_, acetone, 50 °C, 5 h, **259**: 89% in two steps, **269**: 57% in two steps; (**i**) CH_3_CO_2_H, CH_3_CO_2_Na, 100 °C, **260**: 90%, **270**: 61%; (**j**) Bz_2_O, DIPEA, CH_2_Cl_2_, rt, 1 h, 95%; (**k**) *m*CPBA, CH_2_Cl_2_, rt, 0.5 h; (**l**) 60% KOH, EtOH, rt, 0.5 h, 77% in two steps.

To synthesize oxyisocyclointegrin (**264**), *m*-trihydroxy benzene (**250**) was selectively methylated and acylated, forming **252** (Figure 12). Its 2-hydroxy group was selectively protected by treating it with methoxymethyl bromide and DIPEA, affording **253**. Separately, compound **254** was protected with benzyl bromide and hydrolyzed in basic conditions to afford **256**. Subsequently, it was combined with compound **253** in the presence of EDC, producing **257**. Its base-catalyzed BK–VK rearrangement provided **258**, which was alkylated using prenyl bromide to give **259**. Its acid-mediated intramolecular cyclization gave **260**. Subsequently, it was deprotected using Pd (OH)_2_/C-catalyzed hydrogenation in the presence of 1,4-cyclohexadiene (which left the double bond intact), furnishing compound **261**, which was protected again using benzoic anhydride and DIPEA, giving **262**. Its epoxidation using *m*CPBA produced **263**, whose protecting groups were removed via hydrolysis using a 60% KOH solution. The basic conditions also allowed the formation of oxyisocyclointegrin (**264**).

**Figure 13:**
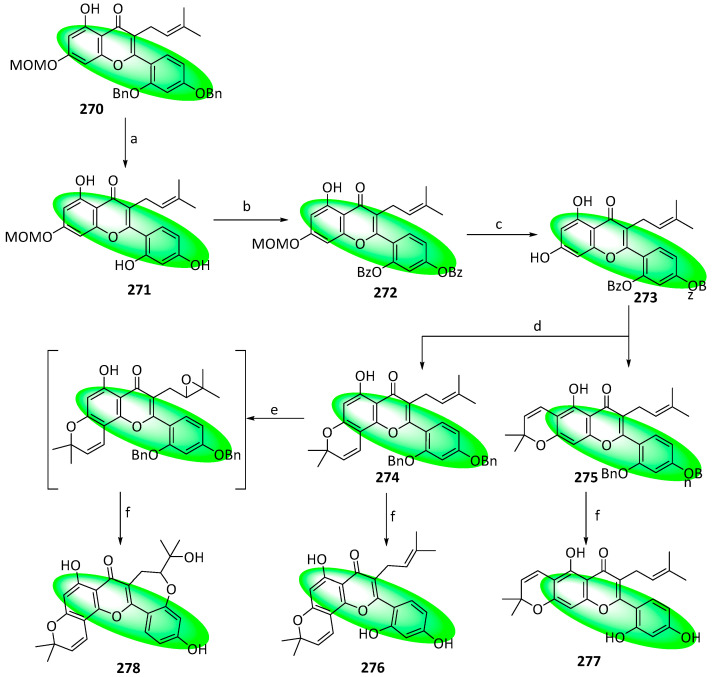
Synthesis of compounds **276** to **278**; (**a**) 20% Pd(OH)_2_-C, 1,4-cyclohexadiene, EtOH, 50 °C, 2 h, 90%; (**b**) Bz_2_O, DIPEA, CH_2_Cl_2_, rt, 1 h, 95%; (**c**) 3M HCl, THF, reflux, 5 h, 78%; (**d**) 1,1-diethoxy-3-methyl-2-butene, 3-methyl-pyridine, xylene, 80 °C, 5 h, **274**: 57%, **275**: 9%; (**e**) *m*CPBA, K_2_CO_3_, CH_2_Cl_2_, 0–5 °C, 2 h; (**f**) 60% KOH, EtOH, rt, 0.5 h, **276**: 88%, **277**: 82%, **278**: 45% in two steps.

To synthesize morusin (**276**), cudraflavone B (**277**), and neocyclomorusin (**278**), compound **250** was treated with acetyl chloride in the presence of AlCl_3_ to deliver compound **265** (Figure 13). Subsequently, by following the same procedure, which transformed compound **252** into compound **260**, compound **265** was transformed into compound **270**, with an overall yield of 14.7%. Similarly, compound **270** was deprotected to furnish compound **271**, which was protected with benzoyl groups to compound **272** with a 95% yield. Additionally, its methoxymethyl group was removed by dilute hydrochloric acid, furnishing compound **273** in 78% yield. An aldol-type condensation utilizing 1,1-diethoxy-3-methyl-2-butene transformed compound 273 into the isomers **274** and **275**, isolated in 68% and 6% yields, respectively. Their 60% KOH solution treatment afforded compounds **276** and **277**, respectively. Finally, the selective epoxidation of compound **274** by *m*CPBA in the presence of K_2_CO_3_ at 0–5 °C and subsequent hydrolysis using KOH produced morusin (**276**) with a yield of 45% over two steps [80]. 

The prenylated isoflavones, 5-deoxy-3′-prenylbiochanin A (**289**) and erysubin F (**299**), and the latter’s novel regioisomer **301**, were synthesized for the first time. Compound **289** has been obtained by hydrolyzing its naturally occurring glucoside or isolating it from *E. sacleuxii* [81,82]. It displays moderate antiviral and antiplasmodial activity [82,83]. Compound **299** has been isolated from many sources, including *E. sacleuxii* [81]. It exhibits moderate antiplasmodial, antibacterial, and PTP1B-inhibitory activity [82,83]. Their synthetic routes use flavanones as key intermediates by treating them with hypervalent iodine-produced isoflavones.

**Figure 14:**
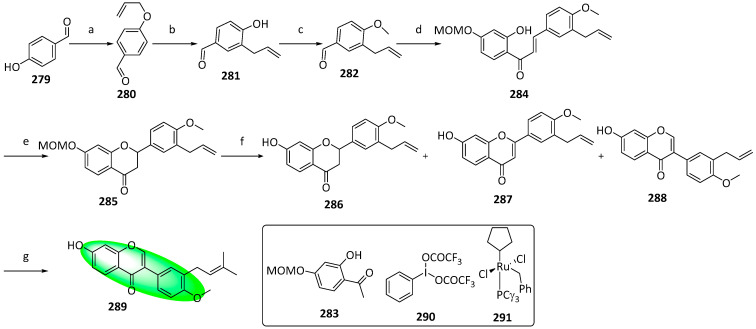
Synthesis of compound **289**; (**a**) K_2_CO_3_, acetone, 65 °C, 91%; (**b**) toluene, microwave irradiation at 250 °C, 84%; (**c**) H_3_Cl, K_2_CO_3_, acetone, 65 °C, 94%; (**d**) **283**, KOH, CH_3_OH, 20 °C, 75%; (**e**) CH_3_CO_2_Na, CH_3_OH, 60 °C, 60%; (**f**) **290**, methyl orthoformate, sulfuric acid; 2. aq. HCl, CH_3_OH, 65 °C, **286**: 16%, **287**: 11%, **288**: 25%; (**g**) **291**, 2-methylbut-2-ene, 5% (mol) THF, 86%.

To synthesize 5-deoxyprenylbiochanin (**289**), compound **279** was allylated and subsequently irradiated with microwaves at 250 °C to yield **281** via Claisen rearrangement (Figure 14). Next, compound **281** was methylated. Then, the product was treated with compound **283**—produced by a regioselective methoxymethyl (MOM)-etherification of 2,4-dihydroxyacetophenone—to give a chalcone, compound **284**, which underwent a base-catalyzed cyclization to afford **285**. Next, compound **285** was converted to **288,** and the other byproducts using a one-pot oxidative rearrangement and deprotection sequence. The byproducts, compounds **286** and **287**, were minimized using a combination of trimethylorthoformate and compound **290**. Finally, compound **288** was converted to **289** using a second-generation Grubbs catalyst (**291**). The final conversion displayed 100% regioselectivity and used tetrahydrofuran (THF) as a solvent due to the insolubility of compound **288** in dichloromethane, the usual choice of solvent for metathesis reactions.

**Figure 15:**
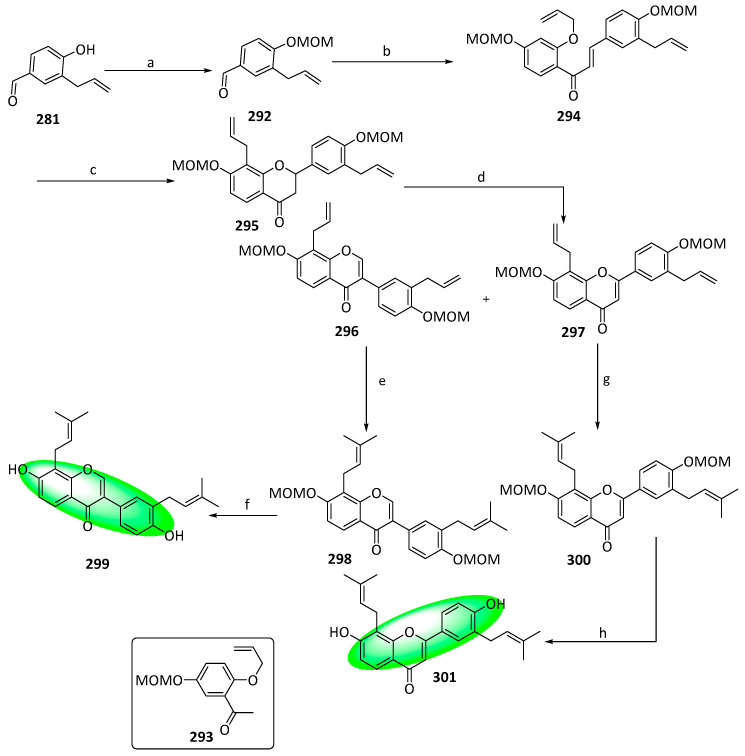
Synthesis of compounds **299** and **301**; (**a**) MOMBr, CH_2_Cl_2_, DIPEA, 82%; (**b**) **293**, KOH, CH_3_OH, 20 °C, 74%; (**c**) 1. toluene, microwave irradiation at 250 °C, 1.5 h; 2. CH_3_CO_2_Na, CH_3_OH, microwave irradiation at 100 °C, 2 h, 50% over two steps; (**d**) **290**, methyl orthoformate, sulfuric acid, **296**: 20%, **297**: 18%; (**e**) **291**, 2-methylbut-2-ene, 5% (mol) CH_2_Cl_2_, 20 °C, 64%; (**f**) aq. HCl, CH_3_OH, 60 °C, 81%; (**g**) **291**, 2-methylbut-2-ene, 5% (mol) CH_2_Cl_2_, 20 °C, 94%; (**h**) aq. HCl, CH_3_OH, 60 °C, 61%.

To synthesize erysubin F (**299**), compound **281** was protected and condensed with compound **293** to yield another chalcone **294** (Figure 15). A microwave-promoted one-pot Claisen rearrangement of compound **294** gave **295**, whose oxidative rearrangement formed regio-isomers, compounds **296** and **297**. Both compounds reacted with 2-methyl-2-butene in dichloromethane to form **298** and **300**, respectively. Finally, both compounds were deprotected to give compounds **299** and **301**, respectively [84]. 

The natural furanoflavonoid glucosides, pongamosides A (**314**), B (**326**), and C (**338**), were synthesized for the first time, with overall yields ranging from 2.9% to 29%. The compounds were isolated from the fruits of *Pongamia pinnata* (L.) Pierre [85]. Other isolates from the same plant exhibited potent anti-inflammatory and analgesic effects, indicating that compounds **314**, **326**, and **338** are also bioactive [86,87]. The synthetic route featured a sodium hydride-promoted BK–VK rearrangement and an acid-catalyzed intramolecular cyclization as key steps. In addition, a phase-transfer-catalyzed glycosylation and Schmidt’s trichloroacetimidate procedure were employed to create the *O*-glycosidic linkage.

**Figure 16:**
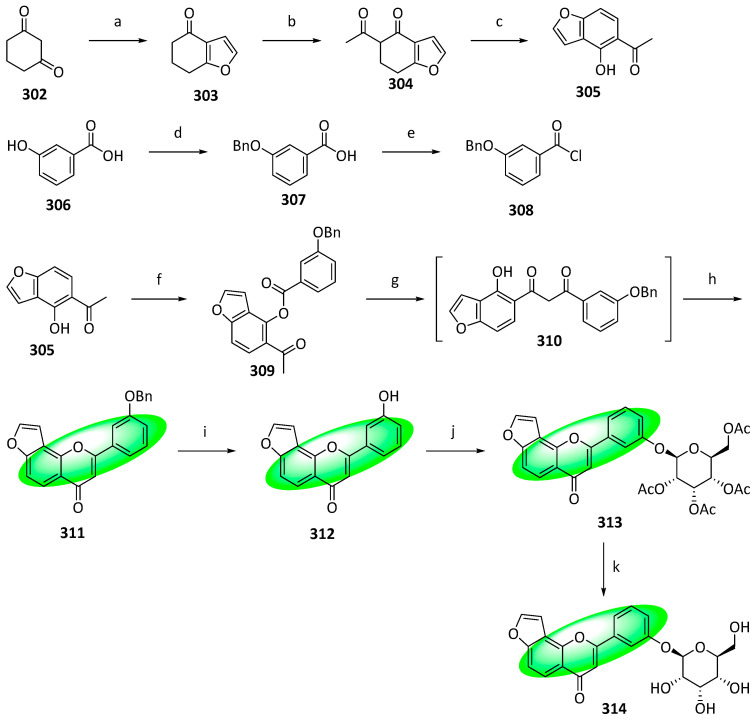
Synthesis of compound **314**; (**a**) chloroacetaldehyde, KI, NaOH, H_2_O, rt, 82%; (**b**) NaH, ethyl acetate, THF, reflux; (**c**) DDQ, toluene, reflux, 68% in two steps; (**d**) BzCl, K_2_CO_3_, DMF, 89%; (**e**) SOCl_2_, pyridine; (**f**) **308**, pyridine, rt, 93%; (**g**) NaH, DMSO, rt; (**h**) CH_3_CO_2_H, HCl, reflux, 89%; (**i**) CH_3_CO_2_H:HCl (2:1), 85 °C, 75%; (**j**) 2,3,4,6-tetracetyl-α-D-glucopyranosyl trichloroacetimidate, 1.1 eq. BF_3_(C_2_H_5_)_2_O, CH_2_Cl_2_, 91%; (**k**) CH_3_OH, CH_3_ONa, rt, 93%.

To synthesize pongamoside A (**314**), compound **302** was treated with chloroacetaldehyde to give compound **303**, which was converted into its enolate form using excess sodium hydride and subsequently reacted with ethyl acetate to produce compound **304** (Figure 16). Finally, it was oxidized by DDQ to compound **305**. Separately, compound **306** was protected and converted into acyl chloride, compound **308**, which was treated with compound **305** to its ester **309**. A BK–VK rearrangement using sodium hydride in DMSO produced **310,** which was converted into **311** using concentrated hydrochloric acid and acetic acid. Similarly, **311** gave **312** by treating with a mixture of hydrochloric acid and acetic acid; **312** was glucosylated using 2,3,4,6-tetracetyl-α-D-glucopyranosyl trichloroacetimidate in the presence of BF_3_ and diethyl ether to yield **313**. Subsequently, it was deprotected using sodium methoxide and methanol and produced pongamoside A (**314**).

**Figure 17:**
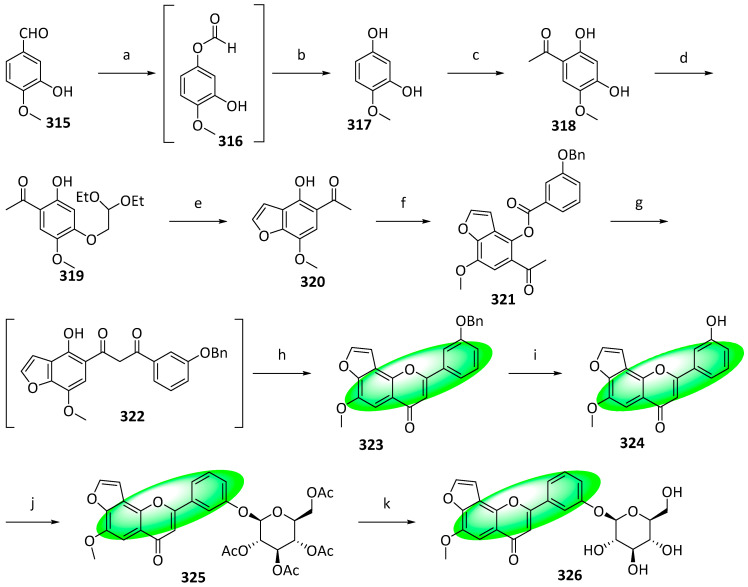
Synthesis of compound **326**; (**a**) SeO_2_, H_2_O_2_, CH_2_Cl_2_, rt; (**b**) K_2_CO_3_, CH_3_OH, rt, 59% in two steps; (**c**) ZnCl_2_, CH_3_CO_2_H, reflux, 66%; (**d**) bromoacetal, DMF, K_2_CO_3_, 80 °C, 67%; (**e**) amberlyst-15, toluene, 120 °C, 89%; (**f**) **308**, pyridine, rt, 83%; (**g**) NaH, DMSO, rt; (**h**) CH_3_CO_2_H, HCl, reflux, 88% in two steps; (**i**) CH_3_CO_2_H:HCl (2:1), 85 °C, 88%; (**j**) 2,3,4,6-tetracetyl-α-d-glucopyranosyl trichloroacetimidate, BF_3_(C_2_H_5_)_2_O, CH_2_Cl_2_, 0 °C, 76%; (**k**) CH_3_OH, CH_3_ONa, rt, 1 h, 85%.

For the synthesis of pongamoside B (**326**), the formyl group of **315** was converted into a formate using Baeyer–Villiger oxidation; subsequent hydrolysis furnished phenol **317** (Figure 17). When it reacted with zinc chloride in acetic acid, it transformed into **318**. Its carbonyl group and C2 phenolic hydrogen formed an intramolecular hydrogen bond, deactivating the C2 phenol and enabling the C4 phenol to react selectively with 2-bromo-1,1-diethoxyethane, affording **319**. The strong acid ion exchange resin Amberlyst-15 promoted the cyclization of **319** and yielded **320**. The transformation of **320** into the final product, **326**, used the same synthetic procedure to convert compound **304** into **314**.

**Figure 18:**
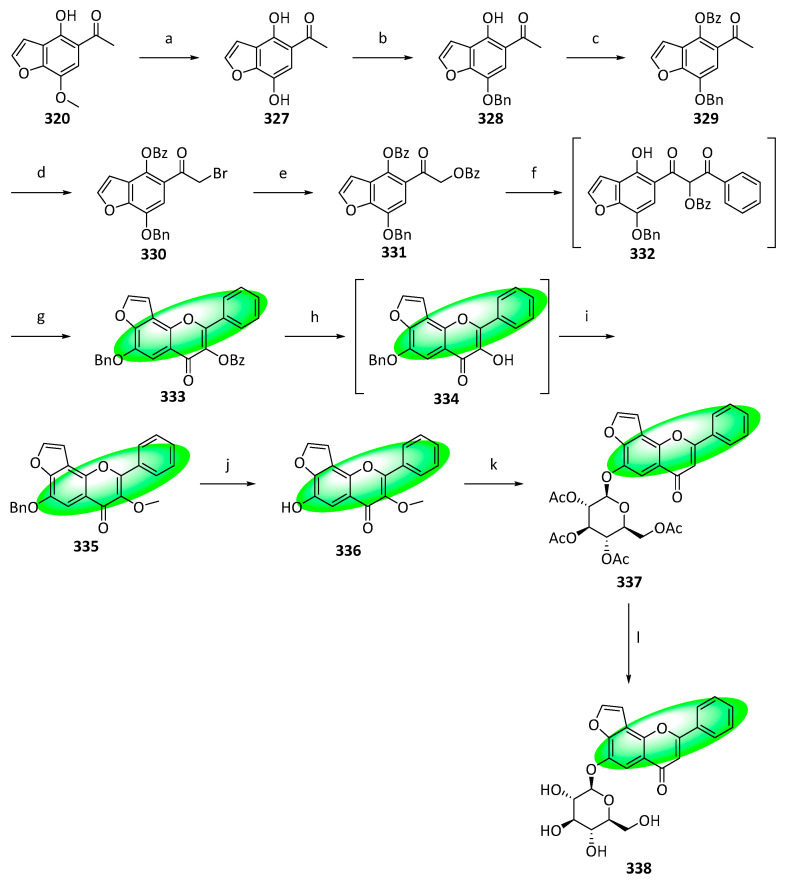
Synthesis of compound **338**; (**a**) BBr_3_, CH_2_Cl_2_, 0 °C to rt, 89%; (**b**) BnBr, K_2_CO_3_, acetone, reflux, 90%; (**c**) BzCl, pyridine, rt, 87%; (**d**) PTT, THF, rt, 88%; (**e**) BzK, CH_3_CN, 80 °C, 82%; (**f**) NaH, DMSO, rt; (**g**) CH_3_CO_2_Na, CH_3_CO_2_H, 100 °C, 61% in two steps; (**h**) NaOH, 60 °C, H_2_O, CH_3_CH_2_OH; (**i**) (CH_3_O)_2_SO_2_, K_2_CO_3_, CH_3_CN, rt, 85% in two steps; (**j**) CH_3_CO_2_H:HCl (5:1), 80 °C, 69%; (**k**) 2,3,4,6-tetracetyl-α-d-glucopyranosyl trichloroacetimidate, BF_3_(C_2_H_5_)_2_O, CH_2_Cl_2_, 0 °C, 79%; (**l**) CH_3_OH, CH_3_ONa, rt, 87%.

To synthesize pongamoside C (**338**), **320** was demethylated using BBr_3_ to avoid selectivity issues later (Figure 18). Subsequently, the C7 phenol was protected using a benzyl group, yielding **328**. The unprotected phenol was protected using a benzoyl group, generating **329**. It was treated with PTT, forming **330**, reacting with potassium benzoate in acetonitrile to yield **331**. A BK–VK rearrangement using sodium hydride in DMSO converted **331** into β-diketone, **332**, which was cyclized utilizing a mixture of sodium acetate and acetic acid, giving **333**. Subsequently, it was treated with sodium hydroxide to form **334**, which was alkylated using dimethyl sulfate to afford **335**. Deprotection of **335** was achieved using hydrochloric acid and acetic acid, yielding the aglycone **336**. The same conditions were used to glycosylate compounds **312** and **314** to transform **336** into **337**. Similarly, the acetyl groups were hydrolyzed using sodium methoxide, producing pongamoside C (**338**) [88]. 

Houttuynoid A (**354**), an antiviral flavone, was synthesized for the first time. The compound was isolated from *Houttuynia cordata* and displayed the most potent inhibitory activity against HSV-1 in the houttuynoid class [89]. Its synthetic route features an I_2_-catalyzed oxa-Michael addition of a chalcone intermediate, thus forming the C6-C3-C6 structure.

**Figure 19:**
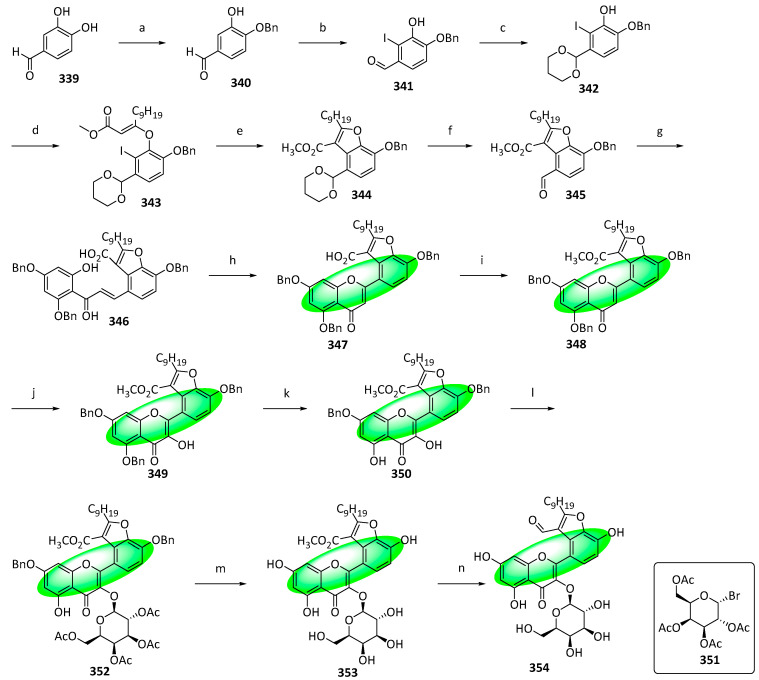
Synthesis of compound **354**; (**a**) BnBr, KI, K_2_CO_3_, acetone, 60 °C, 4 h, 76%; (**b**) ICl, pyridine, 1,4-dioxane, 0 to 18 °C, 48 h, 90%; (**c**) 1,3-propanediol, *p*-TsOH, toluene, 24 h, 82%; (**d**) methyldodec-2-ynoate, K_3_PO_4_, DMF, 110 °C, 16 h, 84%; (**e**) Pd(CH_3_CO_2_)_2_, PPh_3_, Ag_2_CO_3_, DMF, 110 °C, 15 h, 79%; (**f**) HCl, THF/H_2_O, 0 °C, 1.5 h, 90%; (**g**) 1-[2,4-bis(benzyloxy)-6-hydroxyphenyl]ethanone, NaOH, CH_3_CH_2_OH, 50 to 18 °C, 48 h, 98%; (**h**) I_2_, DMSO, 110 °C, 1.5 h, 89%; (**i**) K_2_CO_3_, CH_3_I, DMF, 18 °C, 1.5 h, 99%; (**j**) DMDO, *p*-TsOH, CH_2_Cl_2_/acetone, 18 °C, 18 h, 62%; (**k**) CH_3_CO_2_H/H_2_O, 110 °C, 16 h, 85%; (**l**) **351**, K_2_CO_3_, DMF, 18 °C, 12 h, 99%; (**m**) 1. K_2_CO_3_, CH_3_OH/THF, 18 °C, 1.5 h; 2. H_2_, Pd(OH)_2_, CH_3_OH/THF, 18 °C, 12 h, 98%; (**n**) DIBAL-H. CH_2_Cl_2_, −78 °C, 2 h, 54%.

To synthesize houttuynoid A (**354**), **339** was selectively protected to **340** (Figure 19). It was treated with iodine monochloride to **341**, whose formyl group was protected to **342**. Subsequently, it was reacted with methyl dodec-2-ynoate, giving **343**, which was transformed to **344** using an intramolecular Heck reaction. Hydrolysis of **344** deprotected its formyl group, and a subsequent Claisen–Schmidt condensation with 1-[2,4-bis(benzyloxy)-6-hydroxyphenyl]ethanone yielded **346**. It was converted into **347** using iodine in DMSO, which was methylated at C-2″ using methyl iodide, forming **348**. In situ generated DMDO followed by acid-induced rearrangement oxidized compound **348** to **349**, which was selectively deprotected with acetic acid and water to give **350**. Compound **350** was glucosylated using **351** and gave **352**. It was deacylated and debenzylated via hydrolysis to give **353**, which was reduced using DIBAL-H to afford the final product, houttuynoid A **(354)** [90]. 

Sericetin (**361**), a prenylated flavonol isolated from the root bark of *Mundulea sericea*, can inhibit the growth of various cancer cell lines [91]. It was synthesized in four steps, featuring an electrocyclization to produce the tricyclic core and an aromatic Claisen/Cope rearrangement to incorporate the C8 prenyl group.

**Figure 20:**
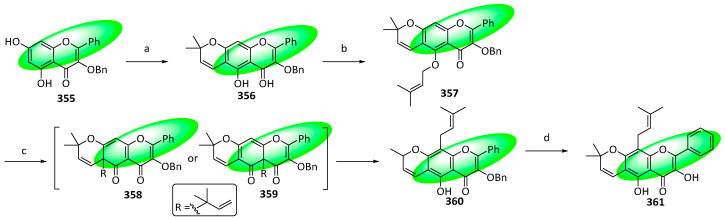
Synthesis of compound **361**; (**a**) 3,3-dimethylacrylaldehyde, Ca(OH)_2_, CH_3_OH, rt, 58 h, 54%; (**b**) 1-chloro-3-methyl-2-butene, K_2_CO_3_, DMF, 40 °C, 14 h, 95%; (**c**) diethylaniline, 270 °C, 4 h, 88%; (**d**) BCl_3_, CH_2_Cl_2_, -78 °C, 2 h, 88%.

To synthesize sericetin (**361**), compound **355**, produced from phloroglucinol using a Friedel–Crafts reaction followed by a pyrone annulation, was cyclized using 3,3-dimethylacrylaldehyde to furnish compound **356** (Figure 20). Subsequently, it was prenylated to **357**, which was treated with diethylaniline in a Claisen rearrangement to give compound **360** via the two intermediate isomers, **358** and **359**. Compound **360** was deprotected to yield the final product, sericetin (**361**) [92]. 

Glaziovianin A (**374**) was first isolated from the leaves of *Ateleia glazioviana* [93]. It exhibited cytotoxicity against various cancer cell lines; its *O*-benzylated derivative inhibited microtubule polymerization more than **374** and colchicine, demonstrating the scaffold’s biological potential [94]. 

**Figure 21:**
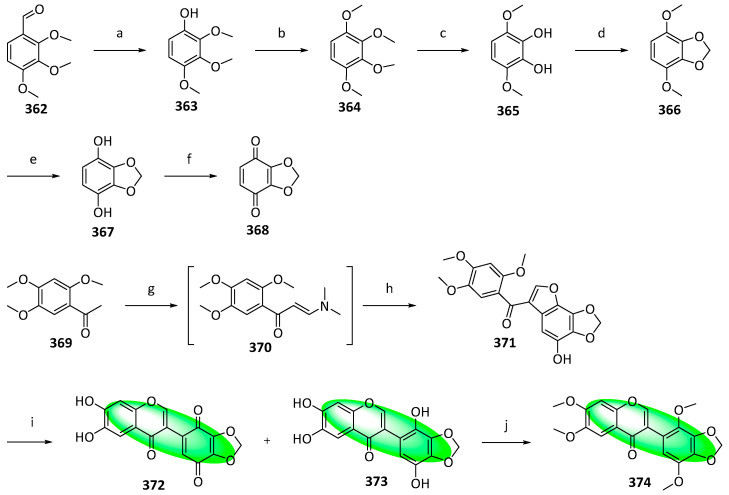
Synthesis of compound **374**; (**a**) CH_2_Cl_2_, CH_3_OH, H_2_O_2_, conc H_2_SO_4_, rt, 3 h, 91%; (**b**) DMF, K_2_CO_3_, CH_3_I, 100 °C, 3 h, 95%; (**c**) CH_2_Cl_2_, AlCl_3_, 0 °C to rt, 18 h 68%; (**d**) DMF, Cs_2_CO_3_, CH_2_I_2_, 100 °C, 6 h, 76%; (**e**) DMF, 98% TMSI, 80 °C, 36 h, 67%; (**f**) CH_2_Cl_2,_ DDQ, rt, 1 h, 91%; (**g**) DMF–DMA, 140 °C, 72 h; (**h**) **368**, CH_3_CO_2_H, rt, 18 h, 45%; (**i**) DMF, TMSI, 80 °C, **372**: 6%, **373**: 26%; (**j**) DMF, K_2_CO_3_, CH_3_I, 100 °C, 6 h, 76%.

To synthesize glaziovianin A (**374**), **362** was transformed into compound **363** using Dakin oxidation (Figure 21). It was then methylated to **364** using methyl iodide and selectively demethylated with aluminum chloride to yield **365**. Subsequently, it was converted into an acetal using methylene iodide to give **366**, which was demethylated using TMSI, furnishing **367**. The oxidation of **367** with DDQ gave **368**. Separately, **369** was condensed with DMF-DMA to **370**, coupled with **368,** which resulted in compound **371**. It was demethylated using excess TMSI, and an intramolecular cyclization gave **372** and its reduced form, **373**. The latter was methylated to glaziovianin A (**374**) [95]. 

### 3.2. Semisynthesis

Analyzing the constituents of the roots of *Derris laxiflora* yielded the potent insecticide isolaxifolin (**380**) [96]. It is also an excellent insect antifeedant as it is not toxic to humans [97]. Apigenin, chosen for its natural abundance, was transformed into **380** with an overall yield of 17%.

**Figure 22:**
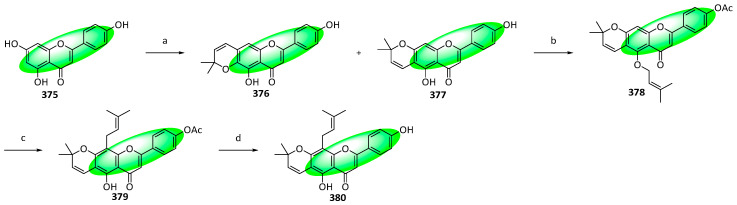
Synthesis of compound **380**; (**a**) 3-methyl-2-butenal, Ca(OH)_2_, CH_3_OH, 18 °C, 72 h, **376**: 15%, **377**: 42%; (**b**) 1. acetic anhydride, pyridine, CH_2_Cl_2_, 18 °C, 6 h; 2. 3-methyl-2-buten-1-ol, DEAD, PPh_3_, THF, 0 to 18 °C, 6 h, 68% in two steps; (**c**) Eu(fod)_3_, CHCl_3_, 60 °C, 6 h, 83%; (**d**) K_2_CO_3_, CH_3_OH, 0 °C, 1 h, 71%.

To synthesize isolaxifolin (**380**), **375** was prenylated using 3-methyl-2-butenal, affording regioisomers **376** and **377** (Figure 22). The latter was treated with acetic anhydride in the presence of pyridine and subsequently condensed with 3-methyl-2-buten-1-ol under Mitsunobu conditions using triphenylphosphine and diethyl azodicarboxylate to give compound **378**. A europium(III)-tris(1,1,1,2,2,3,3-heptafluoro-7,7-dimethyl-4,6-octanediotnae-catalyzed tandem para-Claisen–Cope rearrangement of compound **378** yielded compound **379**, which was hydrolyzed to isolaxifolin (**380**) [98]. 

### 3.3. Synthesis of Derivatives

1-Deoxynojirimycin is an α-glucosidase inhibitor that exhibits anticancer and antidiabetic activities [99,100]. However, its use has been limited due to its poor lipophilicity and susceptibility to degradation. Thus, to improve its pharmacokinetics and antitumor activity, 1-deoxynojirimycin was coupled with kaempferol, affording compounds **387–389**. The derivatives differed in the length of the carbon chain that connected both subunits.

**Figure 23:**
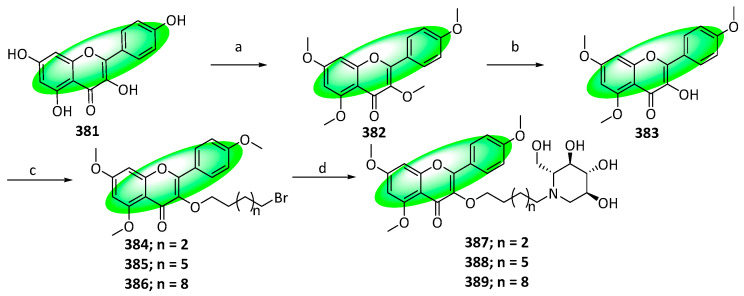
Synthesis of compounds **387–389**; (**a**) dimethyl sulfate, K_2_CO_3_, 50 °C, 14 h, 90%; (**b**) AlBt_3_, r.t, 2 h, 88%; (**c**) K_2_CO_3_, acetone, 60 °C, 12 h, **384**: 1,5-dibromopentane, 75%, **385**: 1,8-dibromooctane, 71%, **386**: 1,11-dibromoundecane, 69%; (**d**) 1-deoxynojirimycin, DMF, 80 °C, 12 h, **387**: 30%, **388**: 23%, **389**: 22%.

To synthesize the derivatives **387–389** of 1-deoxynojirimycin, compound **381** was methylated using dimethyl sulfate and potassium carbonate in acetone, yielding **382**. It was selectively demethylated using aluminum bromide, giving compound 383, which was treated with three linear dibromoalkanes, affording **384** to **386** (Figure 23). Subsequently, treating them with 1-deoxynojirimycin in the presence of potassium carbonate furnished the final derivatives **387** to **389** [101]. 

The major component of the fruits of *Silybum marianum* is silybin, which exerts a myriad of bioactivities, including antioxidant, anticancer, and neuroprotective activities [102,103,104]. Its derivative, 2,3-dehydrosilybin, exhibited more potent cytotoxic and antioxidant activities and was modified further by combining it with a galloyl moiety, an important pharmacophore [105,106,107]. Four derivatives, 3-*O*-galloyl-2,3-dehydrosilybin (**393**), 7-*O*-galloyl-2,3-dehydrosilybin (**396**), 23-*O*-galloyl-2,3-dehydrosilybin (**399**), and 20-*O*-galloyl-2,3-dehydrosilybin (**404**) were synthesized using either chemoselective esterification or Steglich esterification.

**Figure 24:**
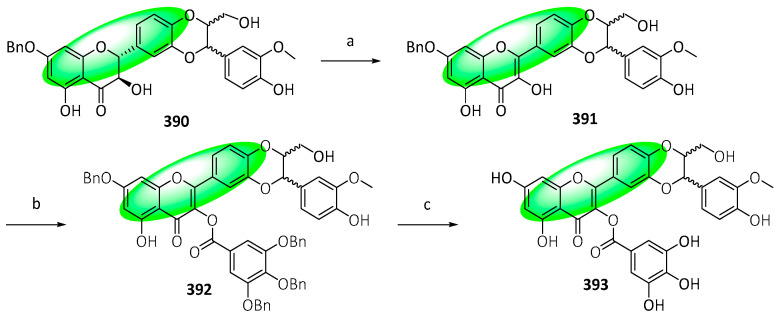
Synthesis of compound **393**; (**a**) 1. I_2_, CH_3_CO_2_H, CH_3_CO_2_K, reflux, 3 h; 2. HCl, CH_3_CH_2_OH, reflux, 3 h; (**b**) DCC, DMAP, CH_2_Cl_2_, rt, 3 h, 30%; (**c**) H_2_-Pd/C, ethyl acetate, rt, 3 h, 70%.

To synthesize 3-*O*-galloyl-2,3-dehydrosilybin (**393**), compound **390** was oxidized using iodine in an acetic acid and potassium acetate buffer, affording **391** (Figure 24). Subsequently, it was condensed with 3,4,5-tri-*O*-benzyl gallic acid using Steglich esterification, forming **392**, which was deprotected to **393**.

**Figure 25:**
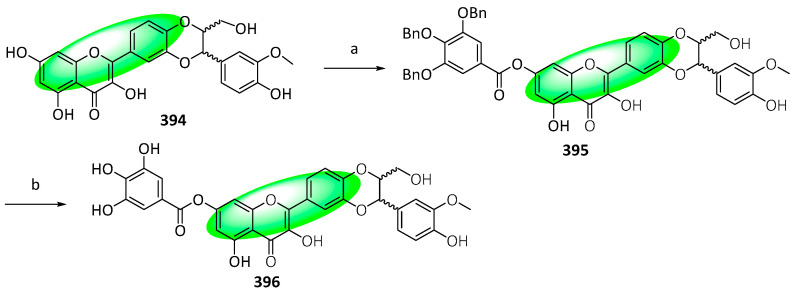
Synthesis of compound **396**; (**a**) 3,4,5-tri-*O*-benzylgalloyl chloride, triethylamine, DCM/CH_3_CN, rt, 1 h, 23%; (**b**) H_2_-Pd/C, ethyl acetate, rt, 12 h, 51%.

To synthesize 7-*O*-galloyl-2,3-dehydrosilybin (**396**), compound **394** was condensed with 3,4,5-tri-*O*-benzylgalloyl chloride in the presence of triethyl amine, forming **395** (Figure 25). Next, its benzoyl groups were removed using hydrogenation in ethyl acetate, yielding **396**.

**Figure 26:**
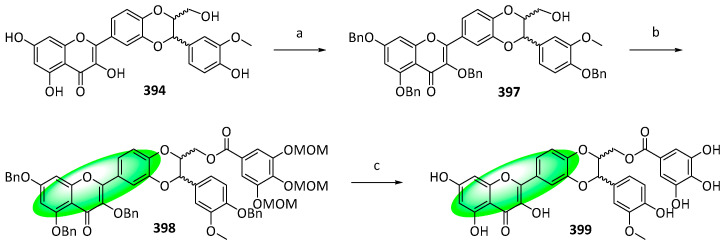
Synthesis of compound **399**; (**a**) BnBr, Cs_2_CO_3_, DMF, 50 °C, 6 h, 61%; (**b**) 1. 3,4,5-tri-O-methoxymethyl gallic acid, DCC/DMAP, CH_2_Cl_2_, rt, 24 h; 2. H_2_-Pd/C, ethyl acetate, rt, 12 h, 73%; (**c**) HCl, CH_3_OH, 3 h, rt, 75%.

To synthesize 23-*O*-galloyl-2,3-dehydrosilybin (**399**), compound **394** was benzoylated to yield **397** (Figure 26). Subsequently, it was galloylated with 3,4,5-tri-*O*-methoxymethylgallic acid in the presence of DCC and DMAP and hydrogenated, delivering **398**. Finally, it was deprotected using hydrochloric acid and methanol to yield **399**.

**Figure 27:**
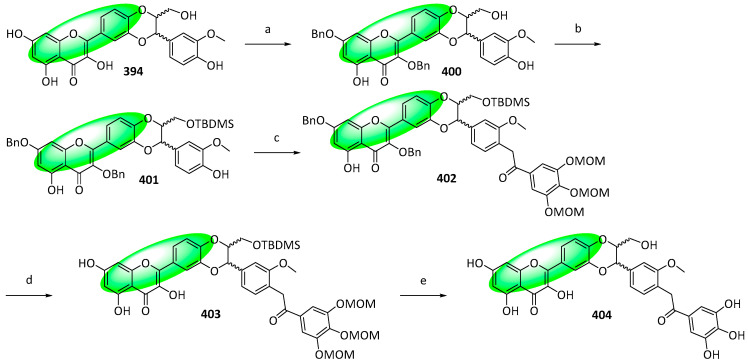
Synthesis of compound **404**; (**a**) BnBr, NaH, DMF, 0 °C to rt, 2 h, 53%; (**b**) TBDMSCl, AgNO_3_, pyridine, 45 °C, 2 h, 41%; (**c**) 3,4,5-tri-*O*-methoxymethylgallic acid, DCC/DMAP, CH_2_Cl_2_, rt, 12 h; (**d**) H_2_-Pd/C, ethyl acetate, rt, 12 h, 51%; (**e**) HCl, CH_3_OH/CH_2_Cl_2_, 3 h, rt, 34%.

To synthesize 20-*O*-galloyl-2,3-dehydrosilybin (**404**), compound **394** was benzylated, yielding compound **400**, which was again protected using a *tert*-butyldimethylsilyl group to compound **401** (Figure 27). Subsequently, it was galloylated using 3,4,5-tri-*O*-methoxymethylgallic acid to form **402**, debenzylated with hydrogen. Finally, an acid-catalyzed removal of the remaining protecting groups transformed compound **403** into compound **404**. [108]. 

### 3.4. Synthetic Methods

In addition to target-oriented and diversity-oriented synthesis, which are covered in the earlier sections, new tools for efficiently preparing or modifying flavonoids or isoflavonoids are also discussed. These tools vary from promiscuous enzymes to synthetic routes/methods of creating novel flavonoids/isoflavonoids.

#### 3.4.1. Enzymes

A new glucuronosyltransferase (UGT), UGT71BD1, was extracted from *Cistanche tubulosa*, a desert herb plant abundant with phenylethanoid glycosides. However, the aglycone analogs were not substrates for the enzyme, which catalyzed the multiglycosylation of phenylethanoid glycosides.

**Figure 28:**
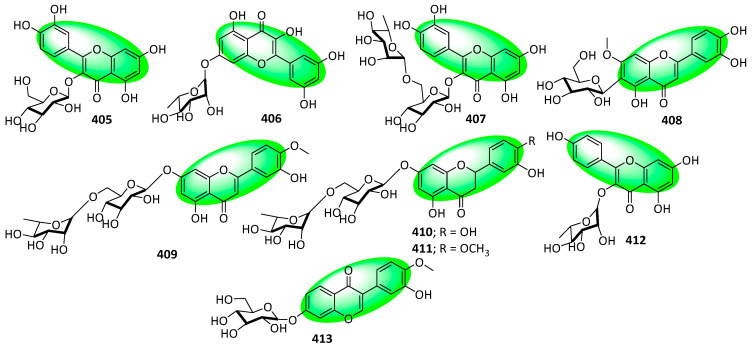
Various substrates for UGT71BD1.

Notably, the enzyme could also accept flavone (**405**, **407–409**, and **412**), flavonol (**406**), flavanones (**410** and **411**), and isoflavone (**413**) glycosides as substrates (Figure 28). UGTs capable of transforming glycoside compounds are rare, but those with such substrate promiscuity are remarkable discoveries. Additionally, the enzyme could also utilize UDP-GlcA as a sugar donor and transfer glucuronic acid to **406** [109]. 

**Figure 29:**
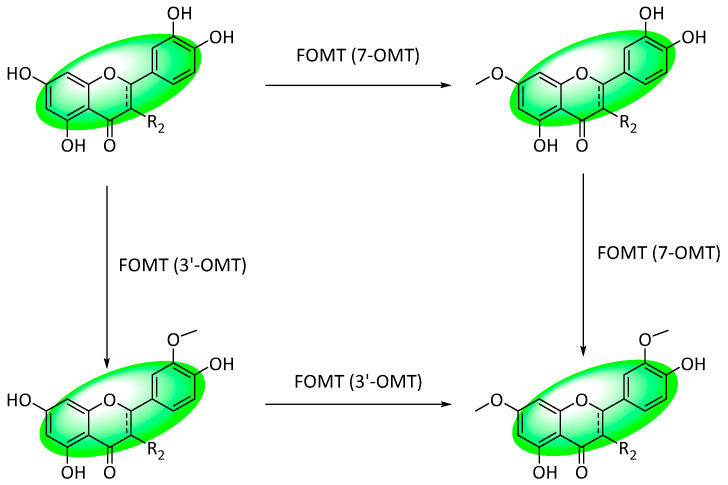
The general catalytic scheme of FOMT.

A new enzyme to generate di-*O*-methylflavonoids in one step was bioengineered; flavonoid *O*-methyltransferase (FOMT) was generated by fusing two *O*-methyltransferases (OMTs), a 3′-OMT (SlOMT3), and a 7-OMT (OsNOMT) (Figure 29).

Using various flavonoids (flavans and their subclasses), the enzymatic activity of FOMT and its constituent enzymes, SlOMT3 and OsNOMT, were tested. SlOMT3 methylated all flavonoids containing a hydroxyl group at C3′ but showed no activity towards isoflavonoids. Furthermore, OsNOMT’s ability to methylate diverse flavonoids was validated. Finally, FOMT was determined to display comparable catalytic activity to its constituting enzymes and could sequentially generate polymethoxyflavonoids [110]. 

#### 3.4.2. Synthetic Methodology

**Figure 30:**
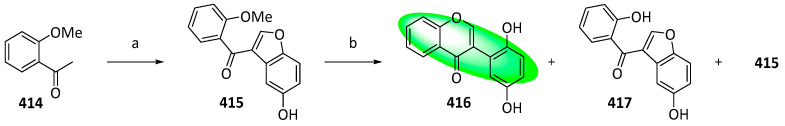
Demethylation of compound **415**; (**a**) 1. DMF–DMA; 2. benzoquinone, CH_3_CO_2_H, 86%; (**b**) BBr_3_, CH_2_Cl_2_, -78 °C, **416**: 11%, **417**: 18%, **415**: 53%.

Methoxybenzoylbenzofuran (**415**) was prepared by treating **414** with DMF-DMA and condensing it with 1,4-benzoquinone in acetic acid (Figure 30). Unexpectedly, the demethylation of **415** produced an isoflavone and a benzofuran, **416** and **417**, respectively. Compound **415** was also recovered. After extensive testing, a general rule was formulated: the demethylation of 3-(2′-methoxybenzoyl)benzo[b]furans produced either 2′,5′-dihydroxyisoflavones, 3-(2′-hydroxybenzoyl)benzo[b]furans, or isoflavone-2′,5′-quinones, depending on the nature and pattern of substituents on the precursor. This rule was used to synthesize compound **374** and reappraise the structures of compounds **458–460** [95]. 

A previously developed method for synthesizing 1,4-benzodioxane neolignans produced 1,4-benzodioxane flavonolignans and flavanolignans. To prove the method’s efficacy and provide sufficient amounts of compounds for biological evaluations, silybin A and B (**426** and **427**), a pair of diastereomeric 1,4-benzodioxane flavonolignans, were synthesized. Additionally, their flavanolignan analogs, isosilandrin A and B (**428** and **429**), were also produced.

**Figure 31:**
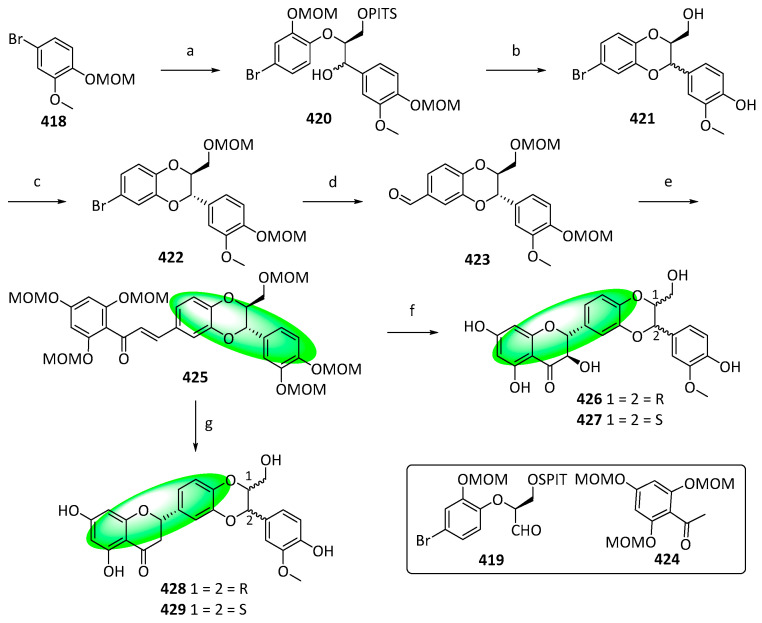
Synthesis of compounds **426** to **429**; (**a**) *t*-BuLi, THF, −78 °C, then **419**, 37%; (**b**) amberlyst 15, toluene, 80 °C, 18 h, 75%; (**c**) MOMCl, DIPEA, CH_2_Cl_2_, rt, 48 h, 96%; (**d**) *t*-BuLi, DMF, THF, −78 °C, 96%; (**e**) **424**, NaOH, CH_3_CH_2_OH, rt, 22 h, 52%; (**f**) 1. H_2_O_2_, NaOH, CH_3_OH; 2. HCl, CH_3_OH, 28%; (**g**) 1. HCl, CH_3_OH; 2. CH_3_CO_2_Na, CH_3_OH, 43%.

A separately prepared aromatic bromide, compound **418**, and aldehyde, compound **419**, were reacted together, affording compound **420** as a diastereomeric mixture (Figure 31). This mixture was deprotected in acidic media, prompting its cyclization and producing another mixture, compound **421**. Protecting the mixture using methoxymethyl chloride yielded only the trans-isomer **422**, which was converted into a lithiate and treated with DMF to introduce a formyl group to form **423**. Subsequently, it was condensed with a protected aldehyde, compound **424**, producing **425**. Epoxidation, deprotection, and subsequent cyclization of **425** yielded diastereomers **426** and **427**. Additionally, first deprotecting compound **425** in acidic conditions and then inducing intramolecular cyclization using basic conditions afforded the diastereomers **428** and **429** [111]. 

A novel method of brominating flavonoids was developed. It employed α,β-dibromohydrocinnamic acid to slowly release bromine at relatively low temperatures, enabling the regioselective bromination of compounds susceptible to oxidation. The method can also be used to brominate flavonolignans, an otherwise arduous task. The equivalents of base (Cs_2_CO_3_ or K_2_CO_3_) used did not exceed 0.5 equivalents, as higher concentrations resulted in the production of hydrogen bromide.

**Figure 32:**
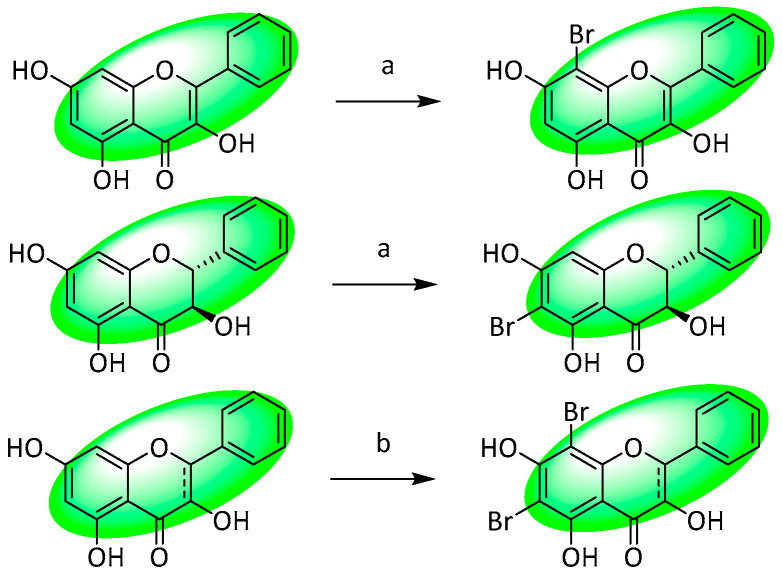
General results of brominating flavonoids; (**a**) α,β-dibromohydrocinnamic acid, Cs_2_CO_3_, DMF, 40 °C, 16 h; (**b**) α,β-dibromohydrocinnamic acid, K_2_CO_3_, DMF, 60 °C, 16 h.

Generally, flavonols containing hydroxy substituents at C5 and C7 were monobrominated at C6, while flavanonols containing both hydroxy groups were monobrominated at C8 only (Figure 32). Changing the base and heating the mixture more strongly usually resulted in dibrominations at C6 and C8. Modifying one of the hydroxyl groups also destroyed regioselectivity, with monobromination occurring at C6 and C8. Interestingly, modifying both hydroxyl substituents restored selectivity (based on the general rule stated above), and removing the C5 hydroxy group entirely (tested with 3,7-dihydroxyflavone) resulted in monobromination at C8 only [112]. 

### 3.5. Reappraisals

The structural complexity of natural products necessitates using different spectroscopic analyses to determine their structure accurately. Unfortunately, due to insufficient data or technical oversights, spectra can be misinterpreted to produce erroneous isolates structures. Revisions of these spectra, prompted by new synthetic discoveries or discrepancies, using contemporary analytical tools yield reappraised structures, rectifying the initial inaccuracies.

**Figure 33:**
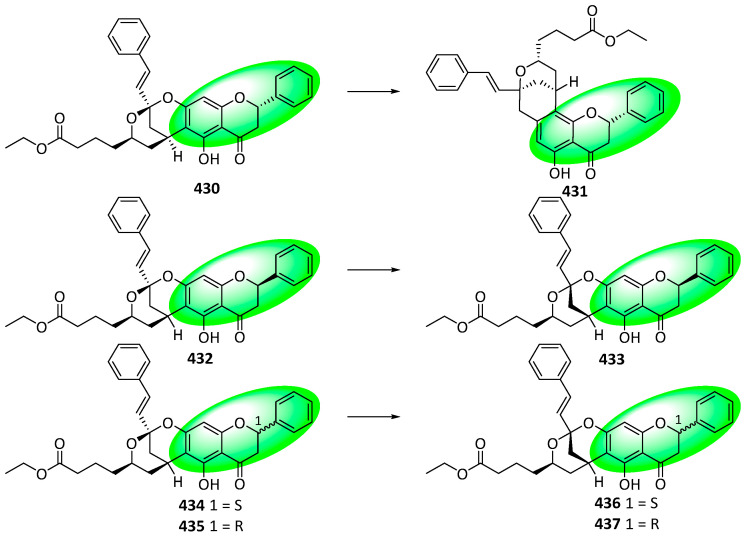
Reappraisal of compounds **430**, **432**, **434**, and **435**.

Elucidating the structures of complex flavanones containing a heteroatomic bicyclononane ring (compounds **130–136**) provided important spectroscopic information, enabling the reappraisal of other compounds having the same motif. The modified compounds were oboflavanone A and B and cryptoflavanones C and D, initially reported as compounds **430**, **432**, **434,** and **435**, respectively (Figure 33). The structure of compound **430** was reappraised as compound **431**. Only the absolute configurations of the other three compounds were modified, producing compounds **433**, **436**, and **437**, respectively [56]. 

**Figure 34:**
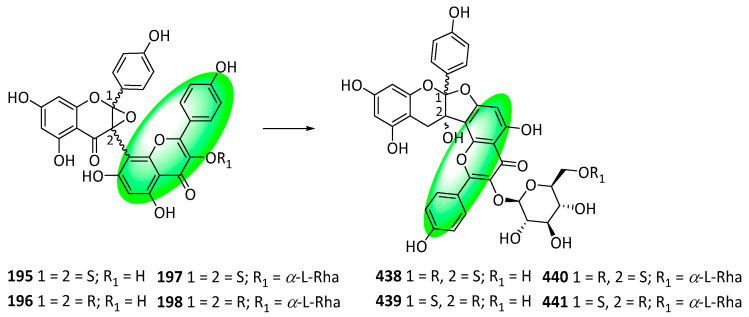
Reappraisal of compounds **195** to **198**.

Interestingly, both the first isolation and subsequent reappraisal of balsamisides A to D (compounds **195–198**) occurred in the last six years (2017 and 2022, respectively). Due to inconsistencies in their ^13^C NMR shift values compared to other analogs, the structures of these compounds were reanalyzed using DP4+ and ECD calculations, yielding the reappraised structures, compounds **438–441**, which contained a fused 3-dihydrofuran ring instead of an epoxide (Figure 34).

**Figure 35:**
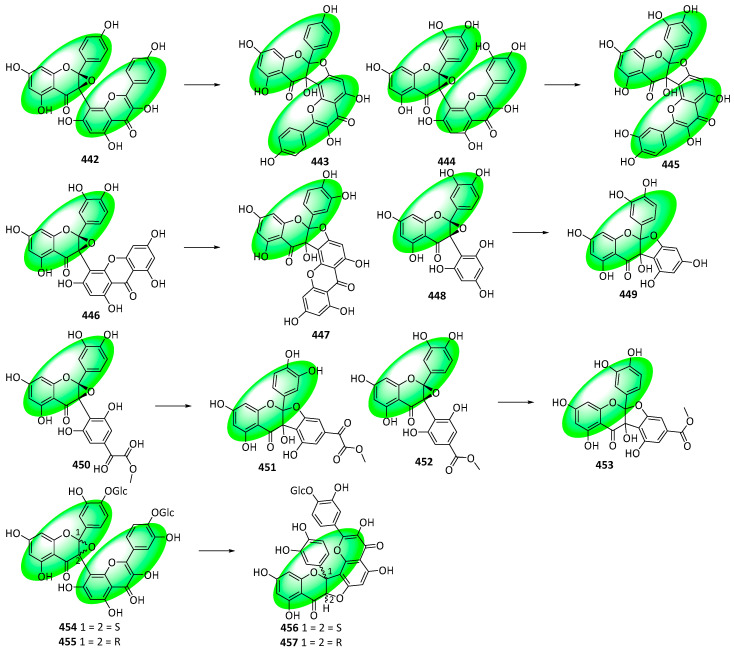
Reappraisal of compounds **442**, **444**, **446**, **448**, **450**, **452**, **454**, and **455**.

Additionally, searching for other flavonoid derivatives reportedly containing a fused epoxide ring at C2 and C3 yielded eight compounds: 3-(kaempferol-8yl)-2,3-epoxyflavanine (**442**), 3-(quercetin-8yl)-2,3-epoxyflavanine (**444**), 3-[4-(1,3,6,8-tetrahydroxyxanthone)]-2,3-epoxyflavanone (**446**), 3-phloroglucinoyl-2,3-epoxyflavanone (**448**), 3-[3-(methylglyoxylate-2,4,6-trihydroxyphenyl)]-2,3-epoxyflavanone (**450**), 3-[3-(1-ethyl ester-2,4,6-trihydroxyphenyl)]-2,3-epoxyflavanone (**452**), and cepabiflas B (**454**) and C (**455**), respectively. Analyzing their ^13^C NMR shift values revealed that none of their structures were accurate; they were reappraised, as shown in Figure 35 [79]. 

**Figure 36:**
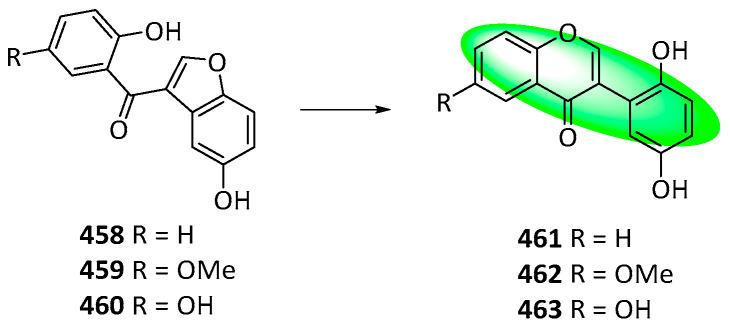
Reappraisal of compounds **458** to **460**.

The demethylation of 3-(2′-methoxybenzoyl) benzo[b]furans produced three similar classes of compounds (as stated in the synthetic rule earlier), making accurate characterizations difficult. Syntheses of 2-unsubstituted benzoylbezofurans with a C2′ hydroxy group were analyzed to determine if they had been correctly identified or erroneously interpreted as the other two classes. This search yielded three SIRT1 inhibitors **458–460**, with discrepancies in their ^13^C NMR spectra. The spectra were reanalyzed, and new structures, **461–463**, were assigned as shown in Figure 36 [95]. 

**Figure 37:**
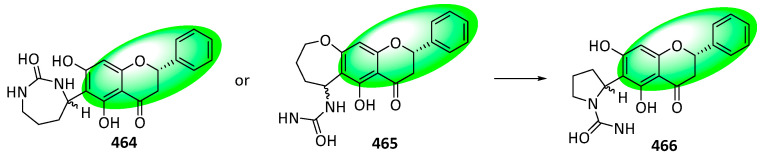
Reappraisal of compounds **464** and **465**.

Aquiledine (**464**) and cheliensisine (**465**) are flavoalkaloids isolated from *Aquilegia ecalcarata* and *Goniothalamus cheliensis*, respectively. Despite having virtually identical ^1^H and ^13^C NMR spectra, both compounds were assigned different structures. However, DFT calculations identified both compounds as identical, and the structure was reappraised as **466**—its stereochemistry remains ambiguous (Figure 37).

**Figure 38:**
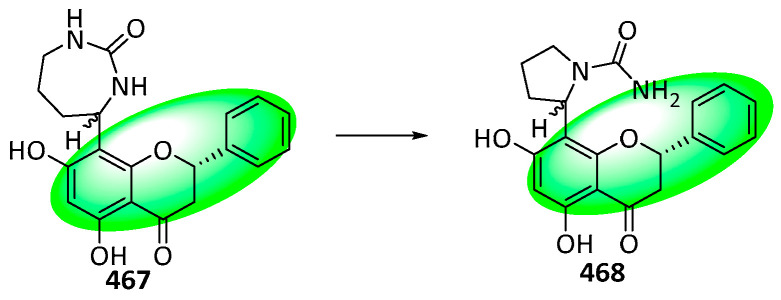
Reappraisal of compound **467**.

Additionally, a regioisomer of compound **464**, isoaquiledine (**467**), was reevaluated similarly to compounds **464** and **465** (Figure 38). The stereochemistry of its new structure, **468**, is also ambiguous [113]. 

## 4. Bioactivities of Flavonoids, Isoflavonoids, and Neoflavonoids

Table 2, Table 3 and Table 4 summarize the bioactivities of the novel compounds discovered or synthesized from 2016 to 2022. However, they do not list all bioactivities exhibited, as some were not expressed using EC_50_s or similar metrics. Instead, they were expressed as percentages at various concentrations or compared to controls and left as is. In addition, it may be due to some compounds’ relative inactivity, which did not merit further extrapolations, calculations, or tests. Thus, additional bioactivities are detailed in the text, along with brief structure–activity relationship studies wherever possible.

From the isolates, compounds **1**, **10**, **27–34**, **55**, **79**, **81**, **148**, **227**, **228**, **248**, and **249** were not tested for unspecified reasons [19,25,31,38,41,60,76,79]. Additionally, compound **82** was not tested due to the putative inactivity of flavanonols in the antigen-induced degranulation assay in RBL-2H3 cells [42]. Similarly, compounds **85**, **87**, **89**, and **90** were not evaluated for their ability to increase glucose uptake [43]. Finally, compounds **103**, **104**, **122**, **124**, **125**, **127** to **129**, **143**, **160**, **161**, **165**, and **178–180** were not tested due to the scarce amounts isolated [50,55,59,62,65].

Compound **2** displayed moderate antioxidant activity: 1.37-fold lower than ascorbic acid (IC_50_: 2.95 µg/mL). Additionally, compound **2** is a potent inducer of apoptosis and could lead to DNA damage [20]. 

Compounds **3** and **4** did not exhibit significant inhibition of leukocyte chemotaxis [21]. Compound **5** was considerably more potent than rutin (IC_50_: 38.6 μM), a powerful antioxidant. Additionally, due to their excellent UV-absorbing capacities, particularly in the UV-A region, compounds **5** and **6** serve dual functions in photoprotection: directly reducing UV radiation transmission and destroying UV-induced reactive oxygen species (ROS) [22]. Compounds **7** and **8** displayed significant cytotoxic activities compared to doxorubicin (IC_50_: 4.47 μg/mL) [23]. Compound **9** showed moderate antioxidant activity, comparable to ascorbic acid (IC_50_: 15 μM) [24]. 

Compounds **11–14** were only evaluated in silico; their inhibition of α-glucosidase and HMG-CoA reductase was evaluated using docking models. All four compounds scored better on α-glucosidase than acarbose, a positive control. Compounds **11**, **12**, and **14** also displayed a better docking score than mevastatin, another positive control, on HMG-CoA reductase. The results indicated that a more significant number of glucosyl moieties decreased bioactivity. For both targets, compound **12** exhibited better scores than compound **11**, and compound **14** displayed better scores than compound **13** [26]. Compounds **15** and **16** showed weak to moderate cytotoxic activities against SiHa and SGC-7901 cells [27]. 

Compound **17** was inactive when tested against B16 and MDA-MB-231 cells. However, another isolate, 5,6,7,3′,4′,5′-hexamethoxyflavone, exhibited cytotoxicity against both cell lines (IC_50_: 14.74 and 34.19 µg/mL, respectively). It indicated that the hydroxyl substituents on 3′ and 4′ of compound **17** were deactivating, as replacing them with methoxy substituents greatly improved bioactivity [28]. 

The protective effects of compounds **18–25** against damage induced by L-glutamate on HT-22 cells were evaluated. Compounds **21** and **22** displayed better activity than trolox, the positive control, between 1.3 to 15 μM. Additionally, compounds **23** and **24** displayed protective activity similar to trolox. Furthermore, the inhibition of ROS generation by compound **21** was evaluated; it decreased ROS generation in a dose-dependent manner. Compounds with more than two sugar groups, such as **19**, **20**, and **25,** were inactive. In contrast, the most potent compounds, **21** and **22,** only contained one sugar group, indicating the group’s deactivating effects, possibly due to steric hindrance. The marginally better protective effects of compound **21** than compound **22** suggest that chirality is not a significant determinant of bioactivity in this scenario [29]. 

The anti-inflammatory activity of compound **26** was evaluated by measuring its inhibition of prostaglandin E2 (PGE2) in LPS-treated RAW 264.7 macrophages [30]. 

Compounds **35–37** were assayed for neuroprotective effects by a cell viability assay on HT22 cells. Compounds **36** and **37** were inactive despite all three compounds differing only by the position and number of acetyl groups on their sugar moieties, suggesting that one of the hydroxyl groups on the saccharides is a major determinant of bioactivity [32]. 

Compounds **38–44** were evaluated for anti-inflammatory effects by measuring the levels of IL-1β, IL-2, IL-6, GM-CSF, and TNF-α in LPS-stimulated PBMCs. The isolates generally showed better anti-inflammatory than ibuprofen, the positive control. Notably, compound **39** inhibited IL-1β, GM-CSF, and TNF-α more substantially than ibuprofen (1.18%, 8.63%, and 0.17% compared to 199.29%, 47.76%, and 12.23%). The percentages were calculated by comparing the levels to the LPS control. Compound **40** inhibited IL-1β, IL-2, and TNF-α more strongly than ibuprofen and considerably upregulated IL-6 production (622.32%) [33]. 

Compounds **45–48** were evaluated for inhibition of PTP1B; compound **48** was inactive, indicating that an unmodified prenyl moiety (present in compounds **45–47**) was necessary for activity. Additionally, the number and length of prenyl chains were also determinants of bioactivity as compounds **46** and **47** inhibited PTP1B more strongly than compound **45** [34]. 

Compounds **49** and **50** displayed moderate antiplasmodial activity but were inactive at inhibiting protein translation [35]. Compound **51** exhibited moderate cytotoxicity, while compound **52** was inactive [36]. 

Compounds **53** and **54** were both moderately active inhibitors of NLRP3 inflammasome, demonstrated by the decrease in IL-1β in the presence of these compounds—0.62 and 0.58 µg/mL, respectively, of IL-1β at 10 µM. In contrast, the positive control, curcumin, inhibited IL-1B levels to 0.28 ug/mL at 50 µM [37]. 

Compounds **56–59** did not inhibit the release of TNF or IL-1β. However, compound **59** strongly inhibited CCL2 secretion by THP-1 cells, demonstrating its usefulness as a selective inhibitor. Additionally, compound **59** contained more methylated hydroxyl groups than the other isolates (although it still contained three unmodified hydroxyl groups), indicating its activating effect. [39]. 

Compounds **60–75** were assayed for their antibacterial activity. However, compounds **68**, **71**, and **74** were inactive. Moreover, the dimerized isoflavones were more potent than their monomeric counterparts (genistein, daidzein MIC > 50 μM). Additionally, the chlorinated biisoflavones, compounds **69**, **70**, **72**, and **73**, showed significant inhibitory activity against *B. subtilis*, *V. parahemolyticus*, and *M. albicansm*, while compound **65** showed excellent activity against MRSA. The latter’s potent activity indicated that the C5′ and C5″ hydroxyl groups (absent in compound **64**) are major determinants of bioactivity. Furthermore, the complete inactivity of compounds **68** and **71** further demonstrated the importance of the C5″ hydroxyl substituent [40]. 

Compounds **76** to **78** and **80** were assessed against multiple bacteria, fungi, and cancer cell lines. However, compound **80** showed no activity, while the active compounds, compounds **76** to **78**, did not inhibit *C. albicans*, *C. neoformans*, or *T. rubrum,* nor did they show any activity towards A-375, HCT-116, and MB-231. Notably, all active compounds showed chain-open prenylation, while none of the compounds with ring-closed isoprene moieties were effective. Additionally, the marginally better antibacterial activity of compound 76 against MRSA indicated that the C3′ and C4′ hydroxyl groups are minor determinants of bioactivity [41]. 

Compounds **83–91** were tested for their inhibitory activity of PTP1B, with compounds **84** and **88** showing the most potency and compound 91 being the most inactive. As IC_50_ data for the other compounds were not determined, inhibitory rates (at 10 μM) were used to quantify and compare activity. Compound **83** displayed slightly lower activity than compound **84**, while compounds **85** and **87** showed considerably weaker activity than compound **84**. Similarly, compounds **86**, **89**, and **90** were slightly weaker PTP1B inhibitors than compound **88**. Additionally, the isolates were also tested for their inhibition of α-glucosidase. All compounds except compound **84** displayed little to no activity. Furthermore, their ability to increase glucose uptake was also evaluated. However, only compound **86** expressed significant activity [43]. 

Compound **92** was tested for its PEDV viral replication inhibition. At 20 μM, 42% of the cells were viable. Hydrogen on C5′ (as in kaempferol 3-O-(2-O-β-D-apiofuranosyl)-α-l-rhamnopyranoside) instead of a hydroxy substituent very slightly boosted inhibition [44]. 

Compound **93** displayed stronger antibacterial activity than streptomycin against *M. flavus* and *P. aeruginosa* (MIC: 344.2 μM for both). It also had lower MIC values than ampicillin against all four bacterial strains. Since compound **93** showed promise as a potent antibacterial, its inhibition of biofilm formation by *P. aeruginosa* was also evaluated. At a concentration of 0.5 MIC, it inhibited 43% of the biofilm formation. Additionally, it also showed weak inhibition (1.8%) at a concentration of 0.125 MIC [45]. 

Compounds **94** and **95** showed no substantial inhibitory activity towards E. coli or MCF-7. Compound **95** was also inactive against *B. subtilis*, implying that its C7 and C4′′′ methoxy groups were deactivating; compound **94** had hydroxy substituents on both positions [46]. 

Compound **96** was inactive when tested for inhibition of tyrosinase. It was evaluated with molecular docking and compared with kojic acid, which had a high docking score despite its two H-bonds. It was attributed to the small distance between the acid and the cupric ion, leading to stronger polar interactions. Compound **96** did not share this trait and had marginally longer polar interactions, which, despite its multiple H-bond interactions with active site residues, led to a lower docking score [47]. 

Compound **97** did not show any protective effects on HEK 293 t-cells damaged by CdCl_2_ [48]. Compounds **98–101** were assessed against five bacteria (MRSA, *E. coli*, *K. pneumoniae*, *A. baumanii*, and *P. aeruginosa*) and two fungi (*C. albicans* and *C. neoformans*). However, compounds **99–101** were inactive, while compound **98** displayed weak activity against *S. aureus*, *A. baumanni*, and *C. neoformans*. Compounds **98** and **99** showed no anthelmintic activity against exsheathed third-stage larvae and fourth-stage larvae of *H. contortus*. The results indicated that the acetyl groups in compounds **99–101** were deactivating [49]. 

Compound **102** was inactive against HepG2 and MCF-7 cells [50]. A bioassay preceded the testing of compounds **105–109**. Consequently, only compounds **105** and **107** were evaluated. Compound **105** was inactive against the fungi *C. krusei* and *C. glabrata*. However, compound **107** showed selectivity for *C. krusei* as it did not inhibit the growth of *C. glabrata* while being more potent than the positive control, fluconazole (IC_50_: 16 μg/mL), at inhibiting *C. krusei*. Furthermore, another isolate, trans-coumaric acid, was also tested against the fungi but showed no activity, demonstrating that the combination of a carboxyethenyl moiety and a flavone core is necessary for activity [51]. 

Compounds **110–111** were screened for their inhibition of *Leishmaniasis donovani* nucleoside hydrolase inhibitors (LdNH). Both were active and were identified as non-competitive inhibitors. Compound **110** was a slightly worse inhibitor; its C5′ hydroxyl group was slightly deactivating [52]. 

Compound **112** was tested against mammalian α-glucosidase and was 2.5 times more active than acarbose (IC_50_: 100 μM), which was used as the positive control [53]. 

Compounds **113–121** were evaluated for inhibiting ROS generation, TNF-α, and IL-8 secretion. Compounds **115**, **117**, and **118** most potently inhibited TNF-α, ROS generation, and IL-8 production. Interestingly, increasing the number of sugar groups did not inhibit bioactivity as compound **119**, containing the least sugar motifs, was also a weaker inhibitor than the other isolates. Furthermore, the C3′ hydroxy group in compound **120** was a better-activating group than the methoxy group in compound **121** [54]. 

Compounds **123** and **126** displayed moderate cytotoxicity, even for the NCI-ADR/RES cell line, which expresses the multidrug-resistant (MDR) phenotype, suggesting that the compounds are not substrates for the Pgp-efflux pump [55]. 

Compounds **130–138** were evaluated for cytotoxicity against HCT-116 cells. However, compounds **130**, **131**, **135**, and **136** were inactive. The unsaturated C20 bond in compound **132** is slightly deactivating compared to **133**, likely due to the restricted movement of the phenyl group [56]. 

Compounds **139** and **140** were evaluated for their antifungal activity. Enantiomerically pure samples of **139** were separately tested, with both isomers possessing identical antifungal potencies. Both compounds inhibited *C. neoformans* more strongly than the positive control, fluconazole (MIC: 25.5 μM) [57]. 

*C. genistoides* is used to prepare green, bitter herbal tea. The tea’s analysis revealed compound **141**, which epimerizes to compound **142** on prolonged heating. Additionally, the bitterness of naringin and compound **141** were tested using descriptive sensory analysis. At the same concentrations, compound **141** was only slightly bitter (7 on a 100-point scale), while naringin was considerably more bitter (26 on the same scale). Since both compound **141** and naringin are structural isomers, the results highlight the importance of sugar positions on the bitterness of the compound [58]. 

Due to the unavailability of enantiopure samples, compounds **144** and **145** were evaluated with their respective enantiomers, gambiriin A1 and A2. Similarly, compounds **146** and **147** were evaluated together. The racemic mixtures were tested for their dental activities, and all three significantly increased the complex modulus of dentin by 3.3-fold. However, the differences in the bioactivity of the enantiomers (if any) could not be determined [60]. 

Compounds **149–155** were tested for their inhibitory activities against NO production in RAW264.7 cells. However, none could inhibit NO production without showing cellular cytotoxicity. Similarly, the isolates did not show appreciable activity on PCSK9 mRNA without damaging the cells [61]. 

Compounds **156–159**, **162–164**, and **166–168** were evaluated for their inhibition of H1N1 neuraminidase (NA). However, they displayed at most 20% inhibition of NA at 20 μM, extremely weak compared to the 55% inhibition exhibited by the positive control (oseltamivir) at 0.1 μM [62]. 

Compound **169** displayed slightly weaker antiviral activity than houttuynoid A (IC_50_: 12.42 μM), another isolate. The two compounds differ only by the substituent on C2″: the former has a houttuynin chain while the latter has a formyl group. A second, bulky chain in compound **169** may increase steric hindrance, slightly decreasing bioactivity [63]. 

Compounds **170–177** exhibited strong to moderate DPPH radical scavenging activities compared to trolox (IC_50_: 23.3 μM), the positive control. The isolates were also evaluated against the parasites *B. bovis*, *B. bigemina*, *B. caballi*, and *T. equi*. However, only compounds **170** and **171** showed any activity. Interestingly, the presence of the bulky galloyl moiety did not significantly deactivate compounds **173** and **175**. The C3 hydroxy group in compound **174** is a major determinant of antioxidant activity as it was considerably more potent than the other analogs. Conversely, it completely inactivated compound **174** against parasites [64]. 

Compound **181** moderately inhibited TNF-α release and reduced phosphorylation levels of p-65-NF-κB at 50 μM. Additionally, it inhibited NF-κB nuclear translocation, demonstrating its inhibition of multiple stages of proinflammatory signaling [65]. 

Compounds **182–184** were evaluated for their xanthine oxidase inhibitory activity, but only **184** showed any activity, albeit very weak [66]. 

Compound **185** did not inhibit α-amylase but strongly inhibited α-glucosidase, being ten times more potent than acarbose, the positive control. Docking studies suggested that it interacts with two key residues, D349 and D214. Water molecules facilitate its interaction with the latter residue [67]. 

Compounds **186–189** were not evaluated in their pure, isolated forms. Instead, the isolated fractions containing these compounds were assayed, and the fractions which contained compound **187** displayed 72–83% inhibition of human hyaluronidase-1 (hHyal-1). Fractions containing compounds **186**, **188**, and **189** showed no activity [68]. 

Compounds **190–194** did not significantly inhibit NO production, tyrosinase, or acetylcholinesterase (AChE) activity. Additionally, only compound **193** showed activity against NF-kB. At 10 μM, compounds **191** and **194** also weakly activated Nrf2 transcription by 1.68-fold and 2.78-fold compared to the control. Similarly, compound **194** inhibited H1N1 by 45% at 10 μM. The compounds were also tested against four cancer cell lines and displayed moderate cytotoxicities at 10 μM: compound **190** inhibited HepG2, SW480, and MCF7 cells by 64%, 45%, and 43%, compound **192** inhibited HepG2, SW480, and MCF7 cells by 44%, 33%, and 37%, compound **193** inhibited SW480 and A549 cells by 33% and 45%, and compound **194** inhibited SW480 cells by 34%. Furthermore, compounds **191** (72%), **193** (55%), and **194** (57%) also moderately inhibited PTP1B at 25 μM [69]. 

Compounds **195–198** weakly inhibited NO production in LPS-Activated BV-2 cells. However, they were inactive against A549, SK-OV-3, SK-MEL-2, and Bt549 cell lines. Compound **195** displayed weak neuroprotective activity gauged by measuring NGF secretion in C6 cells (121%). The other isolates were inactive. Interestingly, the pair of enantiomers, compounds **195** and **196**, had considerably different NO-inhibition potencies, while compounds **197** and **198** (which shared the same core and stereochemistry as compounds **195** and **196**, respectively) had marginally different NO-inhibition potencies [70]. 

Compound **199** was inactive when the HeLa, HepG-2, A549, K562, and HCT-116 cell lines were tested. However, it significantly suppressed IL-6 and IL-1 and upregulated IL-10 expression after LPS stimulation [71]. 

Compounds **200–203** were screened for their inhibition of the superoxide anion (O2-) production and elastase production by human neutrophils when stimulated by fMLP. Compounds **202** and **203** were inactive, whereas compound **201** also inhibited the phosphorylation of JNK and p38 but not ERK [72]. 

Among compounds **204–214**, none inhibited *M. tuberculosis* or *A. baumannii*. The active compounds **204–206** displayed only moderate to weak antibacterial activities. Furthermore, only compound **211** showed any cytotoxicity. Dalbinol, another isolate, was strongly cytotoxic against L5178Y cells (IC_50_: 0.2 μM), indicating that the O3 hydroxy group in compound **208** is strongly deactivating, and methylating it will significantly boost bioactivity [73]. 

Compounds **215** and **216** were evaluated against COX-1, COX-2, and 5-LOX. While compound **216** inhibited COX-1 and COX-2 more weakly compared to ibuprofen (IC_50_: 6.3 μM and 4.2 μM, respectively), it exhibited a higher selectivity ratio towards COX-2 than ibuprofen. Additionally, compound **215** inhibited 5-LOX, unaffected by **216** and ibuprofen. As tomentodiplacone N, another isolate, was inactive towards all three targets, the hydration of Δ1″ of compound **216** is a strongly deactivating modification. Furthermore, adding a methoxy group at C5′ in compound **216**, essentially converting it into compound **215**, traded moderate cyclooxygenase inhibition for potent lipoxygenase inhibition [74]. 

Compounds **217–225** were assayed for their inhibitory effects on NO production in LPS-activated BV-2 cells. However, only compounds **221** and **224** were active. Additionally, the isolates’ neuroprotective effects against Aβ25–35-induced cell toxicity in SH-SY5Y neuroblastoma cells were evaluated, with compound **222** showing significant neuroprotective activity (34.3% increase in cell viability even at 1 μM). Swapping the stereochemistry of C8b and C7b in compound **221**, essentially yielding compound **218**, destroyed bioactivity, emphasizing the importance of stereochemical arrangements in biological systems [75]. 

Compound **226** exhibited moderate antiproliferative activity compared to the positive control, (−)-epigallocatechin gallate (IC_50_: 31.6 μM) [76]. 

Compounds **229–234** did not inhibit insect phenoloxidase, with compound **234** showing no DPPH radical scavenging activity. Additionally, compounds **232–234** did not inhibit tyrosinase either. Furthermore, the tyrosinase inhibitory activities of compounds **229–231** were also extremely weak, comparable only to the inhibitory activity of trolox (IC_50_: 0.7 mM), a positive control. Compounds **230** and **231** showed better bioactivities than their aglycones, compounds **232** and **233**, respectively; a 3-*O*-rhamnopyranosyl moiety is likely an activating group [77]. 

Compounds **235–247** were tested for antibacterial activity against *E. coli*, *S. aureus*, or *S. epidermidis* and antifungal activity against *C. albicans*. The chlorinated derivatives, compounds **235–240**, showed no activity, while only two brominated compounds, **241** and **245**, were active against *H. pylori*. The methoxy group at C4′ in compound **243** is likely a deactivating group (compound **241** had a free hydroxy substituent). Similarly, replacing either of the C6 or C5′ bromine substituents in compound **245** with a methoxy or hydrogen substituent, yielding compounds **246** and **247**, respectively, wholly destroyed bioactivity, suggesting that the bromine groups are activating [78]. 

Compounds **264** and **276–278** were tested against *E.coli*, *S. aureus*, *S. epidermidis*, and *B. subtilis*. However, they were inactive against *E.coli,* and only compounds **276** and **277** exhibited potent antibacterial activity against the other bacteria. As the active compounds contained an isopentane group at C3, and a pyran ring fused to the A ring (motifs absent in compounds **264** and **278**), the substitutions are likely activating groups [80]. 

Compounds **289**, **299**, and **301** were tested against MRSA, *S. enterica* subsp. enterica, *E. coli*, and *C. albicans*. However, all three compounds were inactive against most targets, with only compounds **299** and **301** displaying any activity against MRSA. Interestingly, isoflavone **299** showed slightly better activity than flavone **301** [84]. 

Compound **361** and synthetic intermediate compounds **357** and **360** were tested for their protective effects against cisplatin-induced cytotoxicity in NRK-52E cells at a concentration of 20 μM. Compound 361 decreased cell viability, while compound 360 only showed a marginal increase. Compound **357** significantly increased cell viability, inhibiting ROS generation and reducing cleaved caspase-3 levels [92]. 

Compound **380** was active against five cancer cell lines: 20% cell viability at 17.3, 24.2, 24.7, >30.9, and >34.6 μM against MDA-MB-231, MCF-7, HepG2, LNCaP, and HCT-116 cells, respectively. It arrested cells at the G_0_/G_1_ phase and disrupted the mitochondrial membrane potential (MMP) in MDA-MB-231 cells. Additionally, it did not potently inhibit NO generation in LPS-induced RAW 264.7 cells [98]. 

Compounds **387–389** were assayed for their α-glucosidase inhibitory activity; only compound **387** was less active than 1-deoxynojirimycin (IC_50_: 8.03 μM). However, its lipophilicity (experimentally determined as log P) was more significant than the lipophilicity of 1-deoxynojirimycin (0.49 compared to −0.68). Additionally, the lipophilicity of the more active derivatives, compounds **388** and **389**, was also greater (1.65 and 2.92, respectively), indicating that a longer alkyl chain between the two moieties increased lipophilicity and bioactivity. The compounds were also evaluated for their anticancer activity, but only **388** and **389** showed any activity. Using a wound-healing assay, the ability of compound **389** to effectively inhibit the migration of MCF-7 cells at only 5 and 10 μM was proven. It was also implicated in disrupting DNA synthesis and regulating cell cycle progression. Furthermore, compound **389** could induce MMP collapse and increase intracellular ROS levels, implying that it induced apoptosis through the mitochondrial pathway [101]. 

Compounds **393**, **396**, **399**, and **404** were tested against HUVEC—a model to evaluate antiangiogenic activity—and their cytotoxicity and inhibition of proliferation and migration were measured. Only compound **393** displayed weaker activities than 2,3-dehydrosilybin (IC_50_: 12 μM), indicating that the galloyl group has an activating effect subject to its position [108]. 

While compounds **426–429** were not tested, the more potent bioactivities of synthesized 1,4-benzodioxane lignans than previously reported bioactivities of silybin against Huh7.5.1 cells or HCV demonstrated that the chromanone motif was not necessary for anticancer and antiviral activity [111]. 

## 5. Conclusions

A total of two hundred and forty-nine (249) flavonoids, isoflavonoids, and neoflavonoids were isolated during 2016–2022, along with the flavonoids and isoflavonoids synthesized during those years, representing only a small number of known flavonoids, isoflavonoids, and neoflavonoids. Given their ubiquity in plants and the vast number of plant species yet to be analyzed for their chemical contents, it is plausible to consider the number of undiscovered flavonoids, isoflavonoids, and neoflavonoids as dwarfing their known counterparts. To facilitate the discovery of more potent and structurally diverse analogs, this review collected and summarized relevant information regarding flavonoids, isoflavonoids, and neoflavonoids discovered in the past 7 years to provide interested researchers an accessible guide into the recent developments of the field. It also highlights potent isolates or synthetic derivatives that can serve as lead compounds for future therapeutics. The utility of contemporary computational and spectroscopic methods is demonstrated with the inclusion of reappraisals. Additionally, new methods of synthesizing or modifying flavonoids and isoflavonoids are also discussed to highlight the second aspect of drug development: optimizing the lead.

## Figures and Tables

**Table 1:** Sources and isolation procedures of compounds **1–249**.

Compound Serial	Source	Isolation Method	Ref
**1**	*Strobilanthes kunthianus*	Extracted successively with petroleum ether, chloroform, ethyl acetate, and methanol	[19]
**2**	*Adenosma bracteosum*	Extracted with ethanol; concentrated and partitioned with water, n-hexane, chloroform, ethyl acetate, and n-butanol.	[20]
**3–4**	*Glandularia selloi*	Extracted with methanol	[21]
**5–6**	*Ceratodon purpureus*	Extracted with methanol	[22]
**7–8**	*Afrocarpus gracilior*	Extracted five times with hot 80% methanol; washed with chloroform	[23]
**9**	*Wulfenia amherstiana*	Extracted with ethanol	[24]
**10**	*Lonicera hypoglauca*	Extracted thrice with 85% ethanol; concentrated and extracted with petroleum ether and ethyl acetate	[25]
**11–14**	Fuzhuan brick tea	Extracted with ethanol; concentrated and resuspended in water; washed with petroleum ether	[26]
**15–16**	*Artocarpus nigrifolius*	Extracted with ethanol; concentrated and redissolved in ethyl acetate	[27]
**17**	*Murraya tetramera*	Extracted with methanol	[28]
**18–25**	*Palhinhaea cernua*	Extracted thrice with 75% ethanol; concentrated and resuspended in water; partitioned with petroleum ether, ethyl acetate, and n-butanol	[29]
**26**	*Agastache rugosa*	Extracted with 70% ethanol; concentrated and resuspended in water; partitioned with n-hexane, ethyl acetate, and n-butanol	[30]
**27–34**	*Celmisia viscosa*	Separately extracted with chloroform and ethanol	[31]
**35–37**	*Elsholtzia ciliata*	Extracted with 70% ethanol; concentrated and resuspended in water; partitioned with n-hexane, ethyl acetate, and n-butanol	[32]
**38–44**	*Tephrosia linearis*	Extracted thrice with dichloromethane and methanol; concentrated and partitioned with water and n-hexane; aqueous layer was further partitioned with ethyl acetate	[33]
**45–48**	*Morus nigra*	Extracted thrice with 95% ethanol; concentrated and resuspended in water; partitioned with petroleum ether and ethyl acetate	[34]
**49–50**	*Sphaerocoryne gracilis ssp. Gracilis*	Extracted with methanol	[35]
**51–52**	*Epimedium brevicornum*	Extracted with 95% ethanol	[36]
**53–54**	*Millettia velutina*	Extracted with 95% ethanol; concentrated and resuspended in water; extracted with petroleum ether and ethyl acetate	[37]
**55**	*Onopordum alexandrinum*	Extracted thrice with methanol, concentrated, and partitioned with ethyl acetate and water; the aqueous phase was further partitioned with butanol	[38]
**56–59**	*Ouratea spectabilis*	Extracted with 96% ethanol	[39]
**60–75**	*Streptomyces* sp. HDN154127	Extracted with ethyl acetate	[40]
**76–81**	*Vatairea guianenis aubl.*	Extracted five times with methanol; concentrated and resuspended in water; extracted with dichloromethane and ethyl acetate	[41]
**82**	*Dietes bicolor*	Extracted with 80% methanol; concentrated and resuspended in water and methanol (1:4); partitioned with n-hexane, dichloromethane, and n-butanol	[42]
**83–91**	*Glycyrrhiza uralensis*	Extracted with 90% and 70% ethanol; concentrated and resuspended in water; partitioned with petroleum ether, ethyl acetate, and n-butanol.	[43]
**92**	*Sabia limoniacea*	Extracted with 70% ethanol; concentrated and suspended in water; partitioned with ethyl acetate	[44]
**93**	*Atriplex tatarica*	Extracted with methanol; concentrated and resuspended in water; rinsed with dichloromethane and extracted with n-butanol; concentrated and dissolved in methanol	[45]
**94–95**	*Ochna holstii*	Extracted with methanol and dichloromethane (7:3)	[46]
**96**	*Myrsine africana*	Extracted with methanol; concentrated and resuspended in water; partitioned with petroleum ether, chloroform, ethyl acetate, and n-butanol	[47]
**97**	*Tetraena mongolica*	Extracted thrice with methanol; concentrated and resuspended in water; partitioned with ethyl acetate and n-butanol	[48]
**98–101**	*Drosera magna*	Extracted with methanol and dichloromethane (3:1); concentrated and partitioned with methanol and dichloromethane	[49]
**102–104**	*Phyllanthus acidus*	Extracted with ethanol	[50]
**105–109**	*Mimosa caesalpiniifolia*	Extracted with ethanol and water; concentrated and resuspended in water and ethyl acetate (1:1); extracted with ethyl acetate.	[51]
**110–111**	*Ormosia arborea*	Extracted with ethanol and water	[52]
**112**	*Salvia circinata*	Separately extracted with boiling water and a dichloromethane–methanol (1:1) mixture	[53]
**113–121**	*Corispermum marschallii*	Extracted with acetone–methanol–water (3:1:1); concentrated and partitioned with chloroform, diethyl ether, ethyl acetate, and n-butanol	[54]
**122–129**	Brazilian red propolis	Extracted with 80% ethanol; washed with hexane and concentrated; resuspended in water and ethyl acetate (3:5); partitioned with water and ethyl acetate.	[55]
**130–138**	*Cryptocarya metcalfiana*	Extracted with 95% ethanol; concentrated and resuspended in water; partitioned with ethyl acetate	[56]
**139–140**	*Woodfordia uniflora*	Extracted with ethanol, methanol, 90% methanol, and acetone; concentrated and resuspended in water; partitioned with ethyl acetate and n-butanol	[57]
**141–142**	*Cyclopia genistoides*	Extracted with hot water; concentrated and resuspended in ethanol	[58]
**143**	*Melodorum siamensis*	Dried fruits were extracted with ethyl acetate and methanol; dried leaves were extracted with acetone	[59]
**144–148**	*Pinus massoniana*	Extracted with 70% acetone	[60]
**149–155**	*Sophora tonkinensis*	Extracted with ethanol and water (1:1); concentrated and partitioned with water-saturated n-butanol	[61]
**156–168**	*Pentarhizidium orientale*	Extracted thrice with 80% methanol; concentrated and resuspended in water; partitioned with dichloromethane and n-butanol	[62]
**169**	*Houttuynia cordata*	Extracted twice with ethanol and water (3:2)	[63]
**170–177**	*Saxifraga spinulosa*	Extracted with acetone and water (4:1); concentrated and resuspended in water; extracted with diethyl ether and separately combined with the aqueous phase	[64]
**178–181**	*Mansoa hirsuta*	Extracted with ethanol	[65]
**182–184**	*Berchemia berchemiifolia*	Extracted with methanol; concentrated and resuspended in water; partitioned with chloroform, ethyl acetate, and n-butanol	[66]
**185**	*Arcytophyllum thymifolium*	Extracted with n-hexane, chloroform, and methanol	[67]
**186–189**	*Althaea officinalis*	Extracted with methanol and water (1:1) and centrifuged; the resulting pellet was again extracted with methanol and water (1:1); concentrated and high molecular weight compounds were precipitated by adding the extract into cold ethanol; the suspension was centrifuged, and ethanol was removed from the supernatant	[68]
**190–194**	*Glycyrrhiza uralensis*	Extracted with 95% and 70% ethanol; concentrated and resuspended in water; partitioned with ethyl acetate and n-butanol	[69]
**195–198**	*Impatiens balsamina*	Extracted with 80% methanol; concentrated and resuspended in water; partitioned with n-hexane, chloroform, ethyl acetate, and n-butanol	[70]
**199**	*Carthamus tinctorius*	Extracted with 95% and 70% ethanol; concentrated and resuspended in water; partitioned with petroleum ether and ethyl acetate	[71]
**200–203**	*Daemonorops draco*	Extracted with chloroform; partitioned with water	[72]
**204–214**	*Amorpha fruticosa*	Extracted thrice with dichloromethane and methanol (1:1)	[73]
**215–216**	*Paulownia tomentosa*	Extracted in 96% ethanol; concentrated and resuspended in 10% ethanol; extracted with chloroform; concentrated and resuspended in 90% methanol; washed with hexane	[74]
**217–225**	*Ginkgo biloba*	Extracted with acetone and water	[75]
**226–228**	*Zizyphus jujuba*	Extracted with methanol; concentrated and resuspended in water; partitioned with chloroform, ethyl acetate, and butanol	[76]
**229–234**	*Atraphaxis frutescens*	Extracted with acetone and water (4:1); concentrated and resuspended in water; partitioned with diethyl ether	[77]
**235–247**	*Actinomadura* sp. RB99	Extracted with methanol; concentrated and resuspended in water; partitioned with ethyl acetate	[78]
**248–249**	*Impatiens balsamina*	Extracted with 80% methanol; concentrated and resuspended in water; partitioned with n-hexane, chloroform, ethyl acetate, and n-butanol	[79]

**Table 2:** Cytotoxic and antibacterial activities of the novel compounds.

Compound	Cytotoxic (GI_50_; [Cell Line]; μM)	Ref
**2 ^a^**	4.57 [NCI-H460], 5.67 [HepG2 cells]	[20]
**7 ^a^**	9.02 [Hep-G2]	[23]
**8 ^a^**	15.61 [Hep-G2]	[23]
**15**	48.7 [SiHa], 55.0 [SGC-7901]	[27]
**16**	20.9 [SiHa], 21.3 [SGC-7901]	[27]
**51**	4.3 [HL-60], 7.1 [A-549], 5.4 [MCF-7], 5.1 [SW-480]	[36]
**123**	17.2 [U-251], 27 [MCF7], 19.1 [NCI-ADR/RES], 19.1 [PC-3]	[55]
**126**	25 [U-251], 34.6 [MCF7], 29.9 [NCI-ADR/RES], 21.9 [PC-3]	[55]
**132**	7.2 [HCT-116]	[56]
**133**	6.1 [HCT-116]	[56]
**134**	5.8 [HCT-116]	[56]
**137**	4.2 [HCT-116]	[56]
**138**	4.5 [HCT-116]	[56]
**211**	7.6 [L5178Y]	[73]
**226**	43.5 [HSC-T6]	[76]
**388**	8.8 [HCT-116], 7.6 [A549]	[101]
**389**	3.6 [MCF-7], 7.1 [HCC-1937], 7.7 [HepG-2], 7.8 [HCT-116], 4.5 [BGC-823], 5.1 [A549]	[101]
**393**	18.9 [HUVEC]	[108]
**396**	3.4 [HUVEC]	[108]
**399**	4.3 [HUVEC]	[108]
**404**	11.5 [HUVEC]	[108]
	**Antibacterial (MIC; μM)**	
**9 ^a^**	256 [*S. pneumoniae*], 256 [*B. subtilis*], 128 [*S. aureus*], 512 [*S. flexneri*], 512 [*P. aeruginosa*], 256 [*S. typhi*]	[24]
**60**	6.2 [*B. cereus*], 50.0 [*P. species*], 12.0 [*M. phlei*], 25.0 [*B. subtilis*], 50.0 [*V. parahemolyticus*]	[40]
**61**	12.0 [*B. cereus*], 25.0 [*P. species*], 25.0 [*M. phlei*], 12.0 [*B. subtilis*], 50.0 [*V. parahemolyticus*], 12.0 [MRSA]	[40]
**62**	1.6 [*B. cereus*], 12.0 [*P. species*], 3.1 [*M. phlei*], 6.2 [*B. subtilis*], 6.2 [*V. parahemolyticus*]	[40]
**63**	12.0 [*B. cereus*], 12.0 [*P. species*], 12.0 [*M. phlei*], 12.0 [*B. subtilis*]	[40]
**64**	3.1 [*B. cereus*], 50.0 [*P. species*], 6.2 [*M. phlei*], 6.2 [*B. subtilis*], 12.0 [*V. parahemolyticus*]	[40]
**65**	3.1 [*B. cereus*], 0.8 [*P. species*], 0.8 [*M. phlei*], 0.8 [*B. subtilis*], 0.8 [*V. parahemolyticus*], 0.8 [MRSA]	[40]
**66**	25.0 [*B. cereus*], 25.0 [*M. phlei*], 25.0 [*B. subtilis*]	[40]
**67**	12.0 [*B. cereus*], 12.0 [*P. species*], 12.0 [*M. phlei*], 12.0 [*B. subtilis*], 50.0 [*V. parahemolyticus*]	[40]
**69**	0.4 [*B. subtilis*]	[40]
**70**	0.8 [*B. subtilis*], 3.1 [*V. parahemolyticus*], 1.6 [*M. albicans*]	[40]
**72**	0.8 [*B. subtilis*], 0.8 [*V. parahemolyticus*], 1.6 [*M. albicans*]	[40]
**73**	1.6 [*B. subtilis*], 3.1 [*V. parahemolyticus*], 1.6 [*M. albicans*]	[40]
**75**	1.6 [*V. parahemolyticus*], 1.6 [*M. albicans*]	[40]
**76 ^b^**	29.6 [MRSA]	[41]
**77 ^b^**	37.0 [MRSA], 80.6 [*E. faecium*]	[41]
**78 ^b^**	49.0 [MRSA]	[41]
**93**	295.5 [*M. flavus*], 886.5 [*L. monocytogenes*], 295.5 [*P. aeruginosa*], 591.0 [*E. coli*]	[45]
**94**	14.0 [*B. subtilis*]	[46]
**204**	100 [*S. aureus*], 100 [MRSA], 50 [*E. faecalis*], 100 [*E. faecalis*] ^c^, 100 [*E. faecium*], 100 [*E. faecium*] ^c^	[73]
**205**	100 [S. aureus], 100 [MRSA], 50 [*E. faecalis*], 100 [*E. faecalis*] ^c^, 25 [*E. faecium*], 25 [*E. faecium*] ^c^	[73]
**206**	100 [S. aureus], 100 [MRSA], 25 [*E. faecalis*], 50 [*E. faecalis*] ^c^, 25 [*E. faecium*], 12.5 [*E. faecium*] ^c^	[73]
**241 ^a^**	35.1 [*H. pylori*]	[78]
**245 ^a^**	58.1 [*H. pylori*]	[78]
**264 ^a^**	128 [*S. aureus*]	[80]
**276 ^a^**	8 [*S. aureus*], 8 [*S. epidermidis*], 16 [*B. subtilis*]	[80]
**277 ^a^**	4 [*S. aureus*], 16 [*S. epidermidis*], 16 [*B. subtilis*]	[80]
**278 ^a^**	64 [*S. aureus*], 64 [*B. subtilis*]	[80]
**299**	15.4 [MRSA]	[84]
**301**	20.5 [MRSA]	[84]

^a^ Bioactivities expressed in μg/mL; ^b^ Bioactivities expressed as EC_50_; ^c^ Vancomycin-resistant.

**Table 3:** Antioxidant, anti-inflammatory, antifungal, antiparasitic, and antiviral activities of the novel compounds.

	**Antioxidant (EC_50_; μM)**	
**2 ^a^**	4.04	[20]
**5**	33.5	[22]
**6**	127.8	[22]
**9**	20	[24]
**170**	53.1	[64]
**171**	58.8	[64]
**172**	64.9	[64]
**173**	42.3	[64]
**174**	29.3	[64]
**175**	42.5	[64]
**176**	44.7	[64]
**177**	72.9	[64]
**229**	26.2	[77]
**230**	12.9	[77]
**231**	9.9	[77]
**232**	13.6	[77]
**233**	15.4	[77]
	**Anti-Inflammatory (IC_50_; μM)**	
**26**	16.8 [PGE2]	[30]
**59**	3.1 [CCL2]	[39]
**113**	26.9 [ROS], 66.0 [IL-8]	[54]
**114**	31.5 [ROS], 25.7 [IL-8], 16.3 [TNF-α]	[54]
**115**	5.7 [ROS], 28.9 [IL-8], 9.3 [TNF-α]	[54]
**116**	5.7 [ROS], 25.5 [IL-8]	[54]
**117**	5.4 [ROS], 24.5 [IL-8], 9.7 [TNF-α]	[54]
**118**	7.1 [ROS], 7.2 [IL-8], 12.8 [TNF-α]	[54]
**119**	38.5 [ROS], 51.9 [IL-8], 16.3 [TNF-α]	[54]
**120**	36.6 [ROS], 46.7 [IL-8], 23.2 [TNF-α]	[54]
**121**	6.0 [ROS], 20.7 [TNF-α]	[54]
**181**	19.3 [TNF-α]	[65]
**193**	24.6 [NF-kB]	[69]
**200**	3.1 [O_2_^−^ inhibition], 4.5 [elastase inhibition]	[72]
**201**	1.3 [O_2_^−^ inhibition], 3.1 [elastase inhibition]	[72]
	**Antifungal (MIC; μM)**	
**9 ^a^**	256 [*T. longifusis*], 256 [*A. flavus*], 512 [*M. canis*], 512 [*F. solani*], 128 [*C. albicans*], 256 [*C. glabrata*]	[24]
**107 ^a^**	15 [*C. krusei*]	[51]
**139**	1.9 [*C. neoformans*], 14.8 [*C. albicans*]	[57]
**140**	10 [*C. neoformans*]	[57]
	**Antiparasitic (EC_50_; μM)**	
**110**	28.2 [LdNH]	[52]
**111**	25.6 [LdNH]	[52]
**170**	9.4 [*B. bovisa*], 19.9 [*B. bigeminaa*]	[64]
**171**	12.1 [*B. bovisa*], 22.7 [*B. bigeminaa*]	[64]
	**Antiviral (EC_50_; μM)**	
**169**	17.72 [HSV]	[63]

^a^ Bioactivities expressed in μg/mL.

**Table 4:** Miscellaneous activities of the novel compounds.

	**Inhibition of NO Production (IC_50_; μM)**	
**195**	33.33	[70]
**196**	56.86	[70]
**197**	39.16	[70]
**198**	31.02	[70]
**221**	2.91	[75]
**224**	17.23	[75]
	**Inhibition of PTP1B (IC_50_; μM)**	
**45**	12.5	[34]
**46**	7.7	[34]
**47**	5.3	[34]
**84**	5.9	[43]
**88**	6.7	[43]
	**Inhibition of Tyrosinase (IC_50_; mM)**	
**229**	0.9	[77]
**230**	4.7	[77]
**231**	1.2	[77]
	**Inhibition of α-glucosidase (IC_50_; μM)**	
**84**	20.1	[43]
**112**	39	[53]
**185**	28.1	[67]
**387**	40.56	[101]
**388**	1.78	[101]
**389**	0.24	[101]
	**Inhibition of Oxygenases (IC_50_; μM)**	
**215**	0.37 [5-LOX]	[74]
**216**	26.3 [COX-1], 9.5 [COX-2]	[74]
	**Neuroprotective Activity (EC_50_; μM)**	
**35**	69.7 [HT22]	[32]
	**Inhibition of Xanthine Oxidase (IC_50_; μM)**	
**184**	95.4	[66]
	**Glucose uptake rate in an insulin resistant HepG2 cell model**	
**86**	95%	[43]

## Data Availability

Not applicable.

## Abbreviations

DIPEA	*N*,*N*-Diisopropylethylamine
EDC	1-Ethyl-3-(3-dimethylaminopropyl)carbodiimide
*m*CPBA	*meta*-Chloroperoxybenzoic acid
DDQ	2,3-Dichloro-5,6-dicyano-1,4-benzoquinone
DMSO	Dimethyl sulfoxide
PTT	Phenyltrimethylammonium tribromide
HSV-1	Herpes simplex virus–1
DMDO	Dimethyldioxirane
DIBAL-H	Diisobutylaluminium hydride
TMSI	Trimethylsilyl iodide
DMF-DMA	N,N-Dimethylformamide dimethyl acetal
DCC	N,N′-Dicyclohexylcarbodiimide
DMAP	4-Dimethylaminopyridine
